# Supplementary material for: Study on the Synthesis, Antioxidant Properties, and Self-Assembly of Carotenoid–Flavonoid Conjugates
Source: Molecules. 2020 Feb 1;25(3):636. doi: 10.3390/molecules25030636 (PMC7038153; doi:10.3390/molecules25030636)
Supplement: Supplementary file 1 [file molecules-25-00636-s001.pdf]

molecules

MDPI

Supplementary material for the article:

Study on the synthesis, antioxidant properties, and self-assembly of carotenoid-flavonoid conjugates

Ildikó Línzembold [1], Dalma Czett [1], Katalin Böddi [1], Tibor Kurtán [2], Sándor Balázs Király [2], Gergely Gulyás-Fekete [1], Anikó Takátsy [1], Tamás Lóránd [1], József Deli [1,3], Attila Agócs [1] and Veronika Nagy [1,*]

[1] University of Pécs, Medical School, Department of Biochemistry and Medical Chemistry, Szigeti út 12, H-7624 Pécs, Hungary
[2] University of Debrecen, Department of Organic Chemistry, POB 400, H-4002, Debrecen, Hungary
[3] University of Pécs, Faculty of Pharmacy, Department of Pharmacognosy, Rókus u. 2, H-7624 Pécs, Hungary
* Correspondence: vera.nagy@aok.pte.hu; Tel.: +36-72-536-001 (31864 ext.)

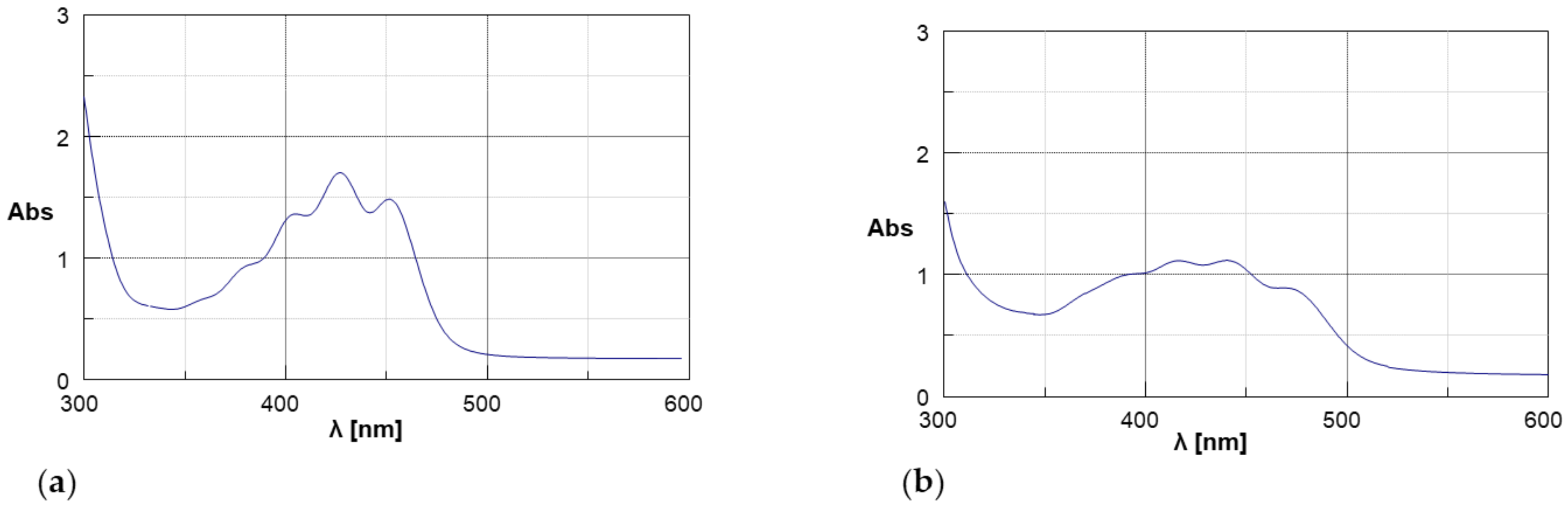

Figure S1. UV-vis spectra of daidzein-8′-apo-β-carotenol conjugate **(16)** in (**a**) acetone/ethanol 1:2; (**b**) acetone/ethanol/water 1:1:2.

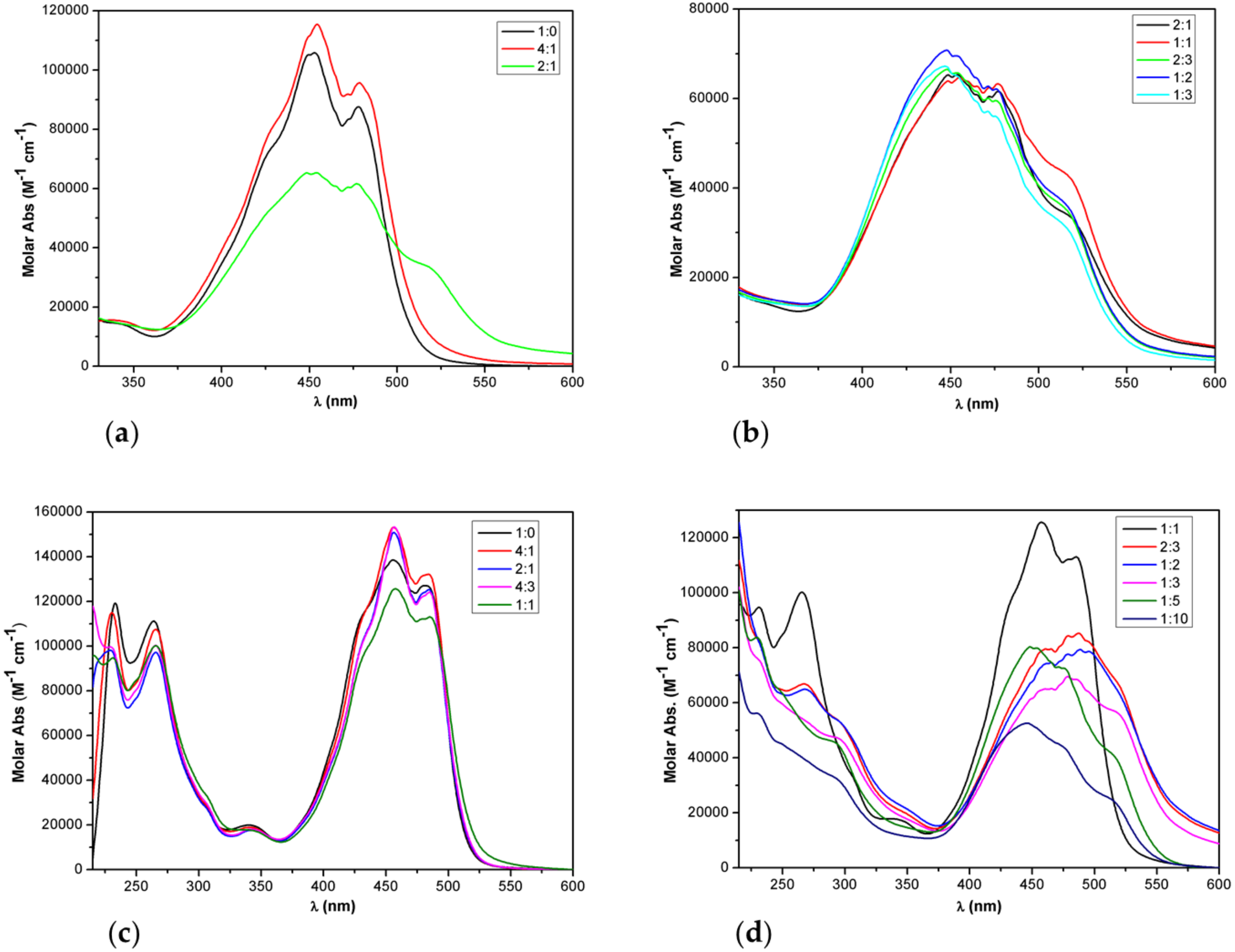

Figure S2. UV-vis spectra of ditriazole daidzein-zeaxanthin conjugate **(17)** in (**a**) acetone/water 1:0-2:1; (**b**) acetone/water 2:1-1:3 (**c**) THF/water 1:0-1:1. (**d**) THF/water 1:1-1:10.

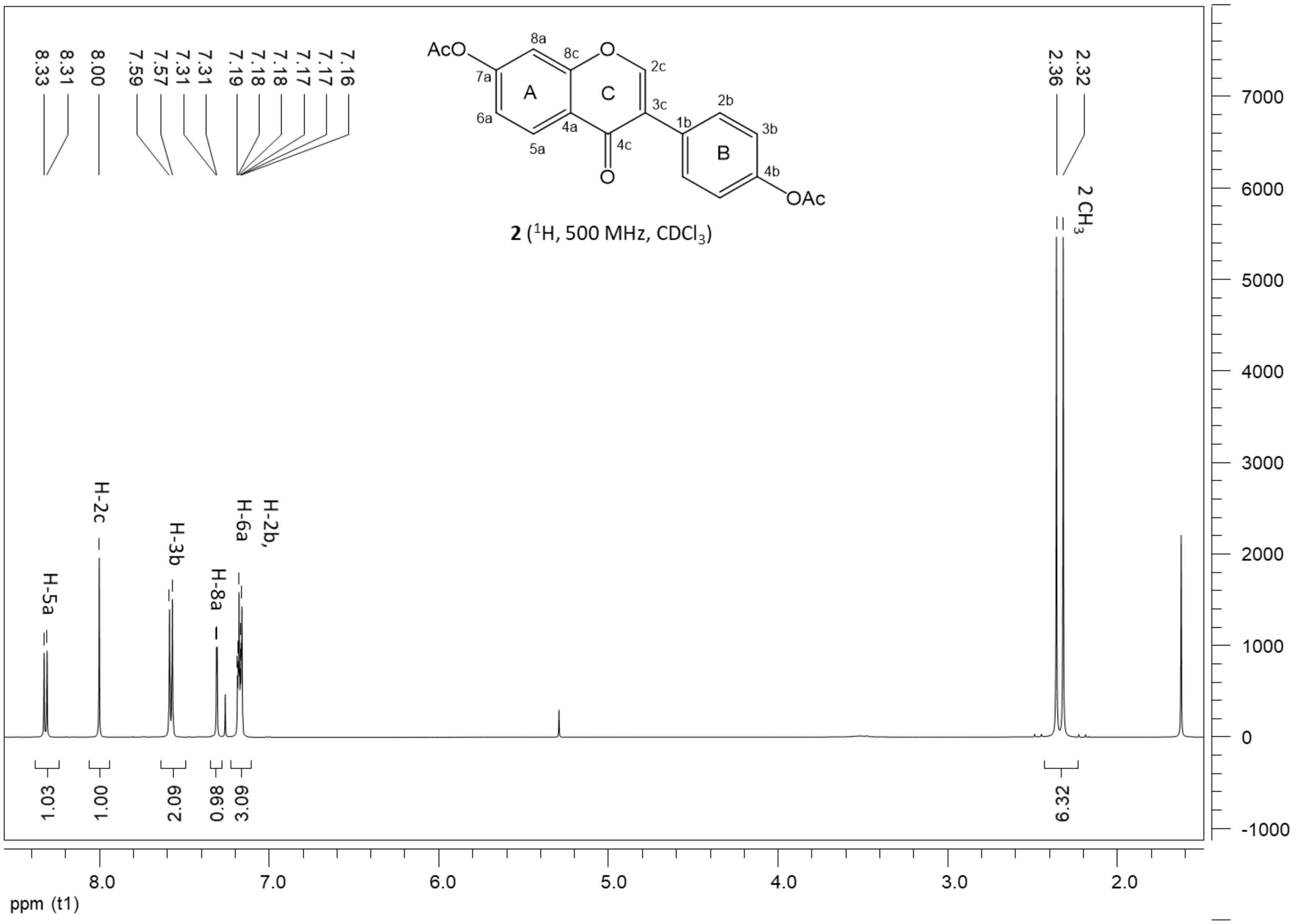

Figure S3. ^{1}H NMR spectrum of diacetyl daidzein (**2**)

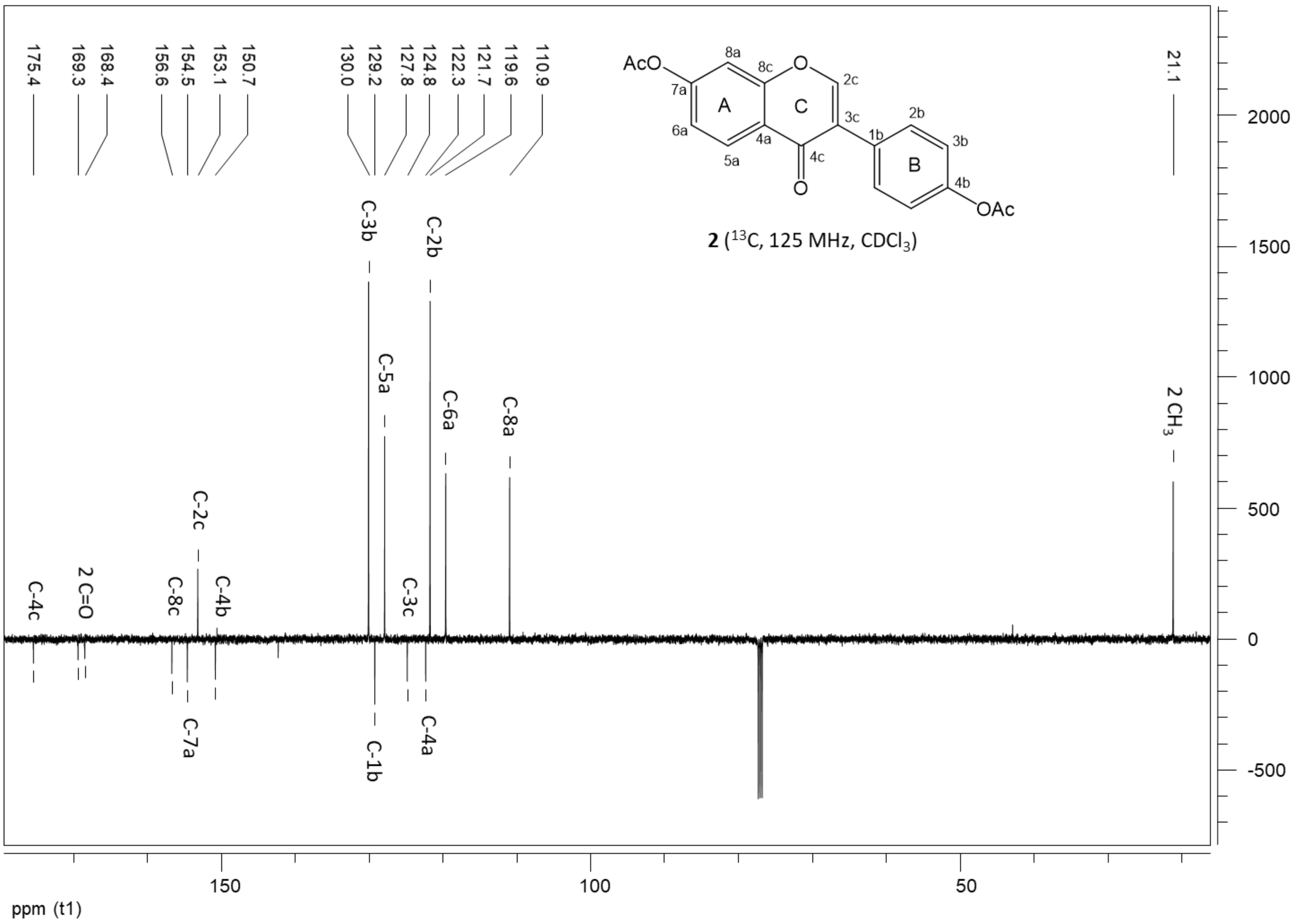

Figure S4. ^{13}C-apt NMR spectrum of diacetyl daidzein (**2**)

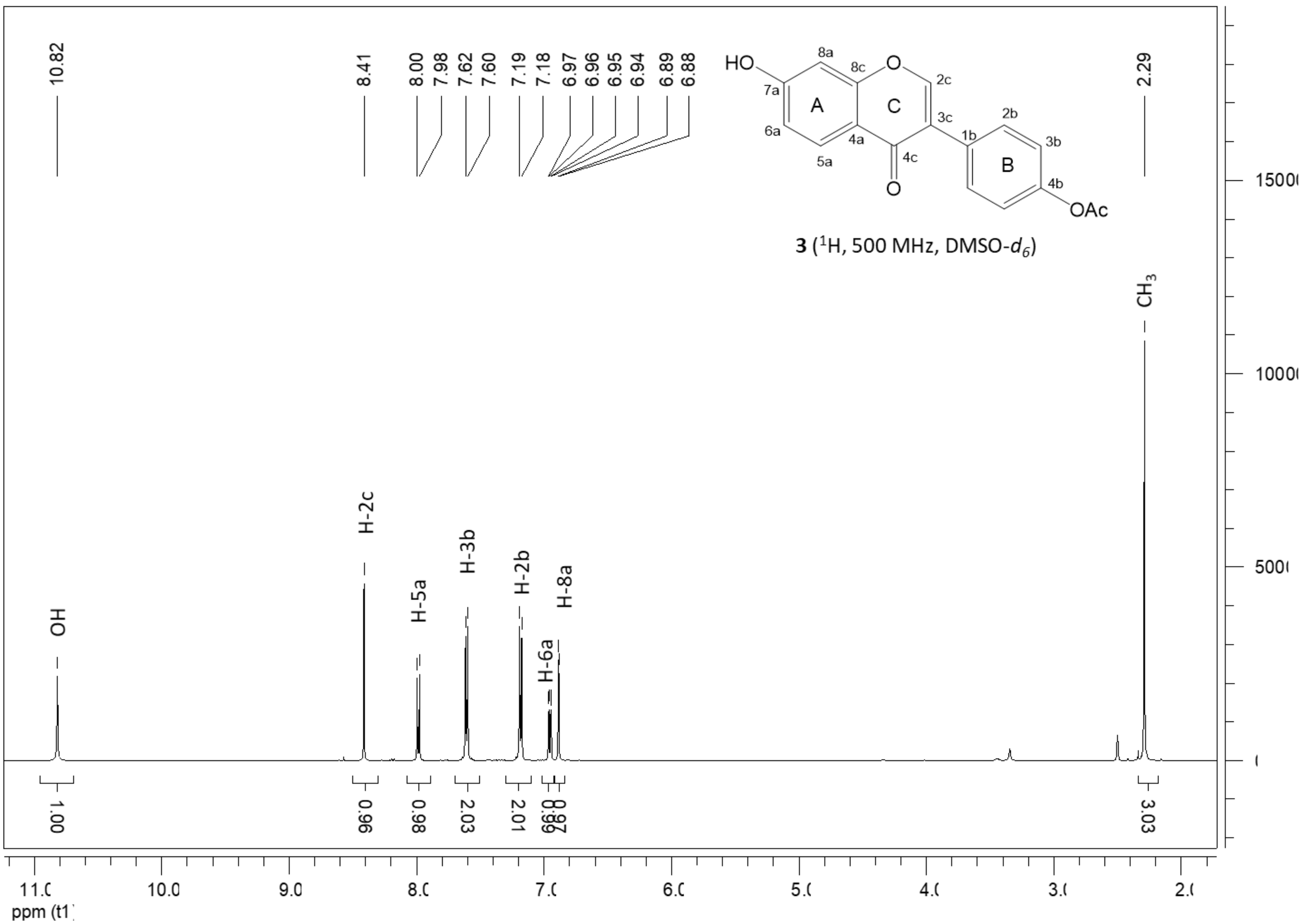

Figure S5. ^{1}H NMR spectrum of 4-acetyldaidzein (**3**)

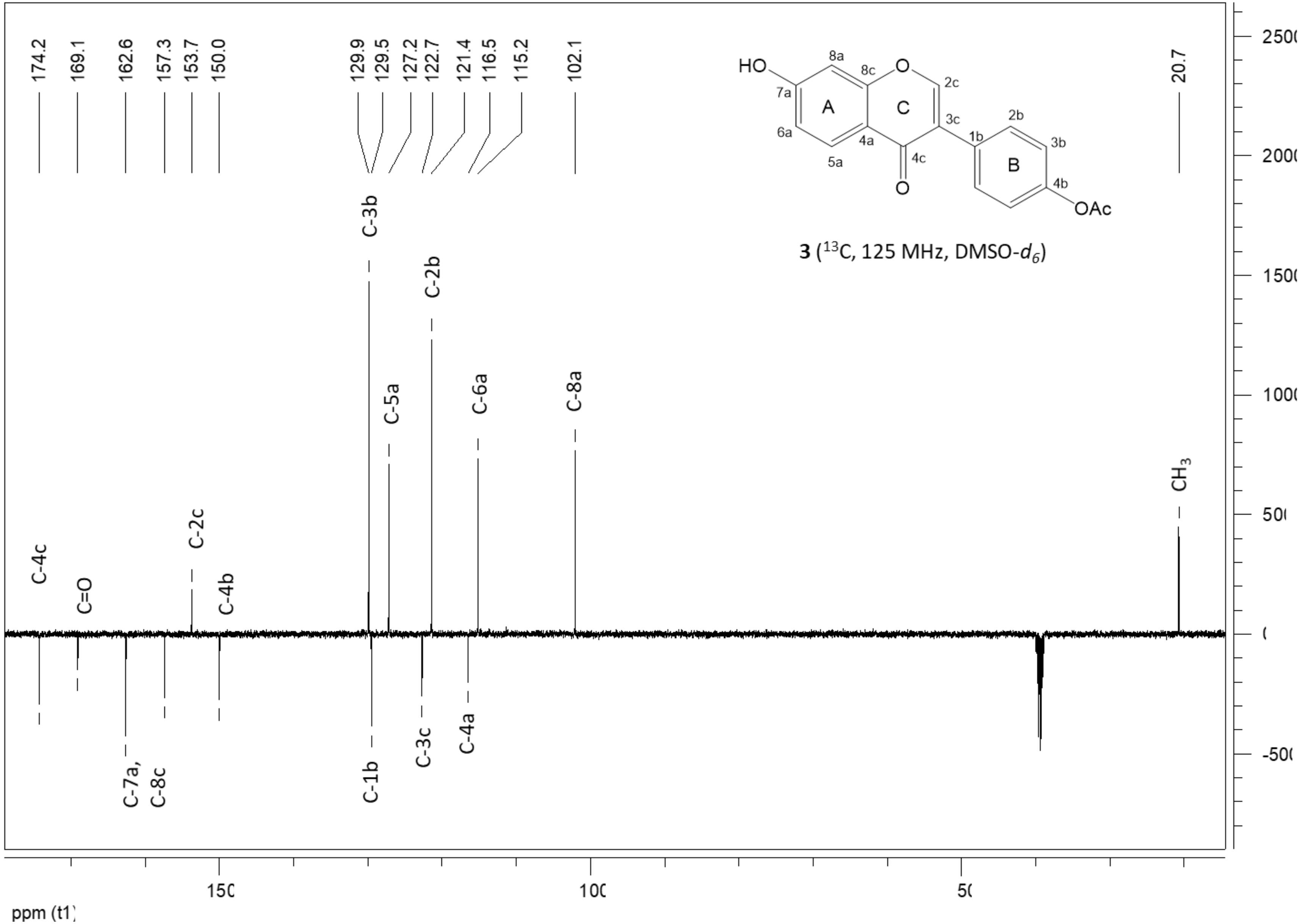

Figure S6. ^{13}C-apt NMR spectrum of 4-acetyldaidzein (**3**)

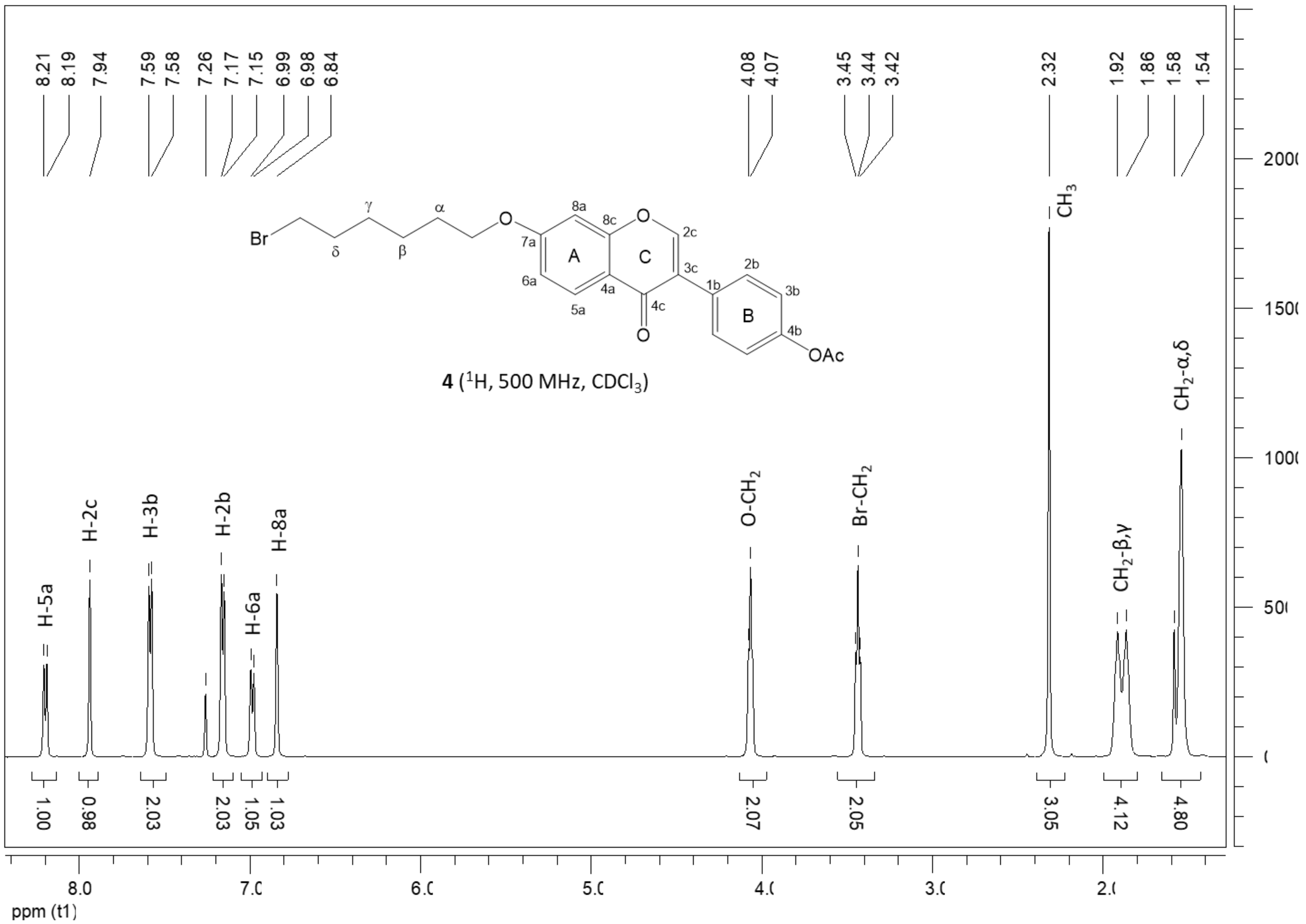

Figure S7. ^{1}H NMR spectrum of 7-bromohexyl-4-acetyldaidzein (**4**)

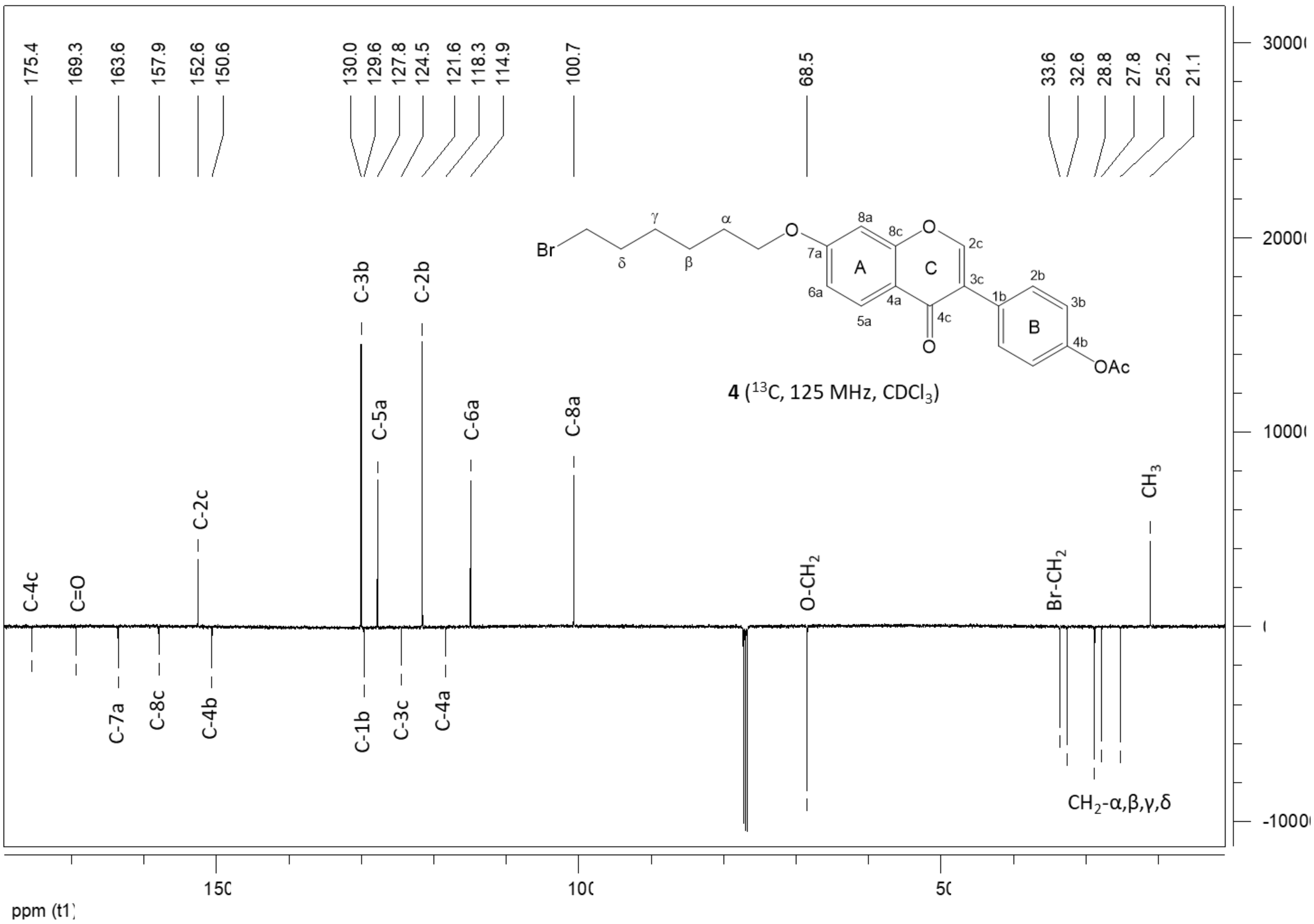

Figure S8. ^{13}C-apt NMR spectrum of 7-bromohexyl-4-acetyldaidzein (**4**)

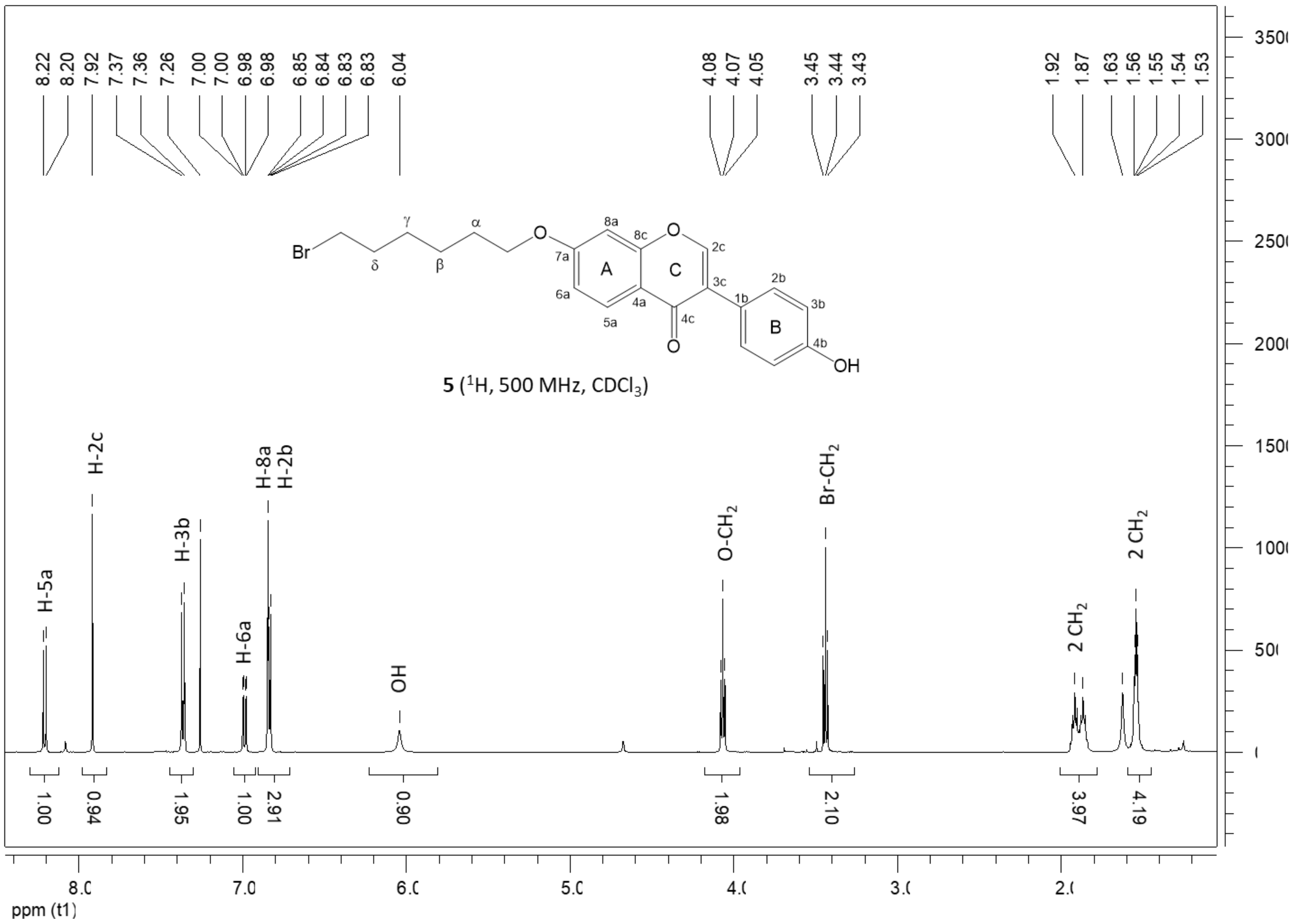

Figure S9. ^{1}H NMR spectrum of 7-bromohexyl-daidzein (**5**)

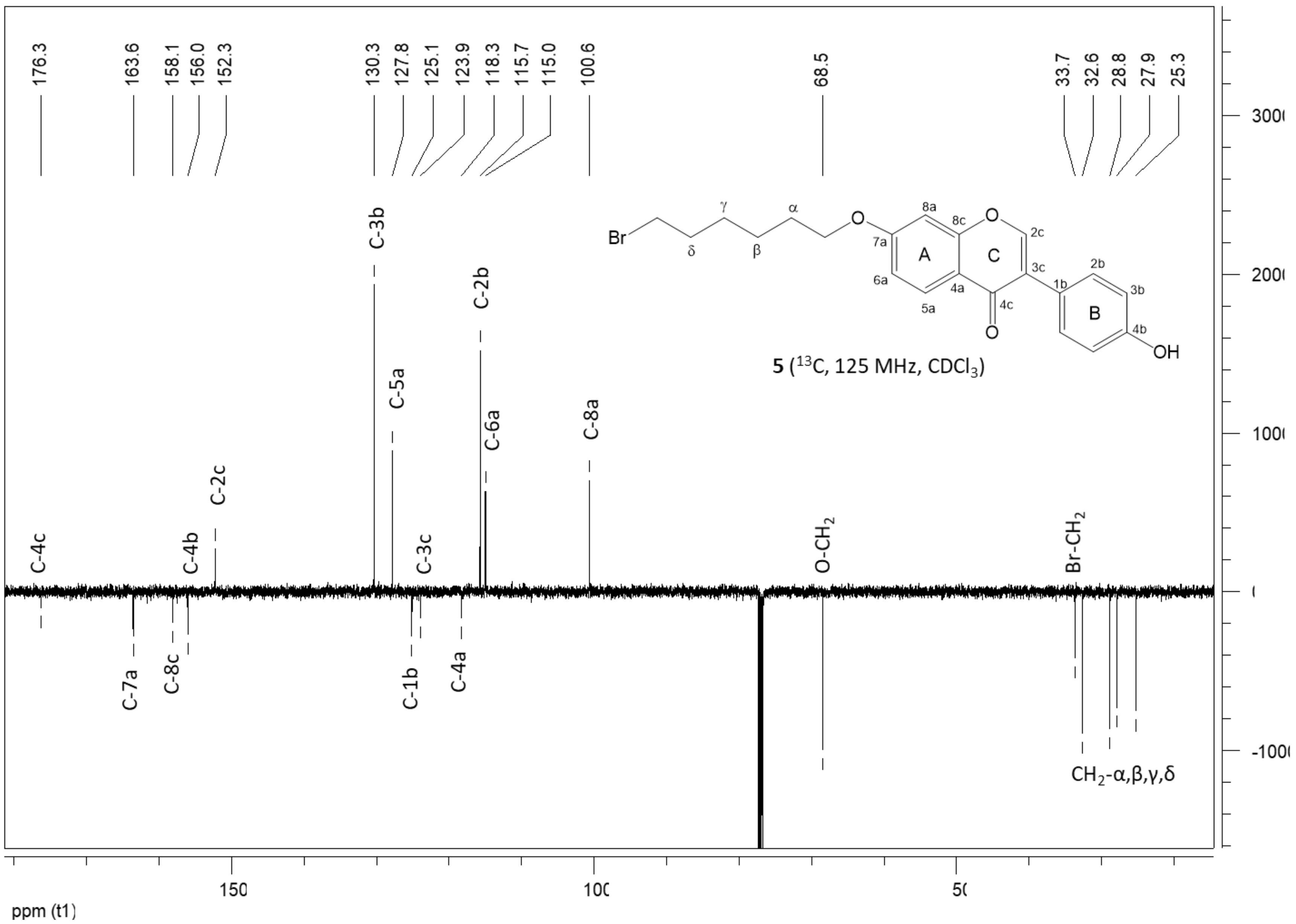

Figure S10. ^{13}C-apt NMR spectrum of 7-bromohexyl-daidzein (**5**)

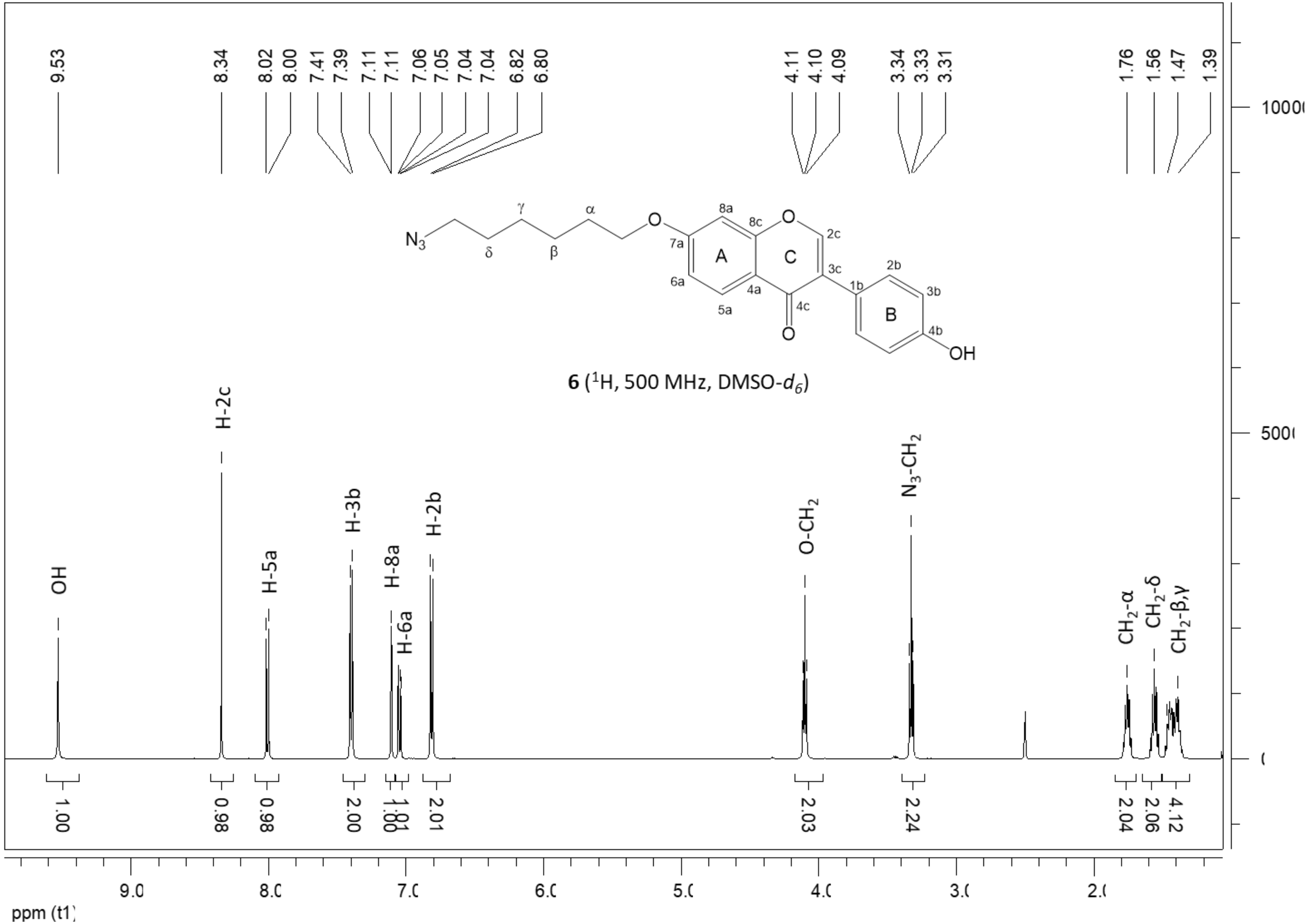

Figure S11. ^{1}H NMR spectrum of 7-azidohexyl-daidzein (**6**)

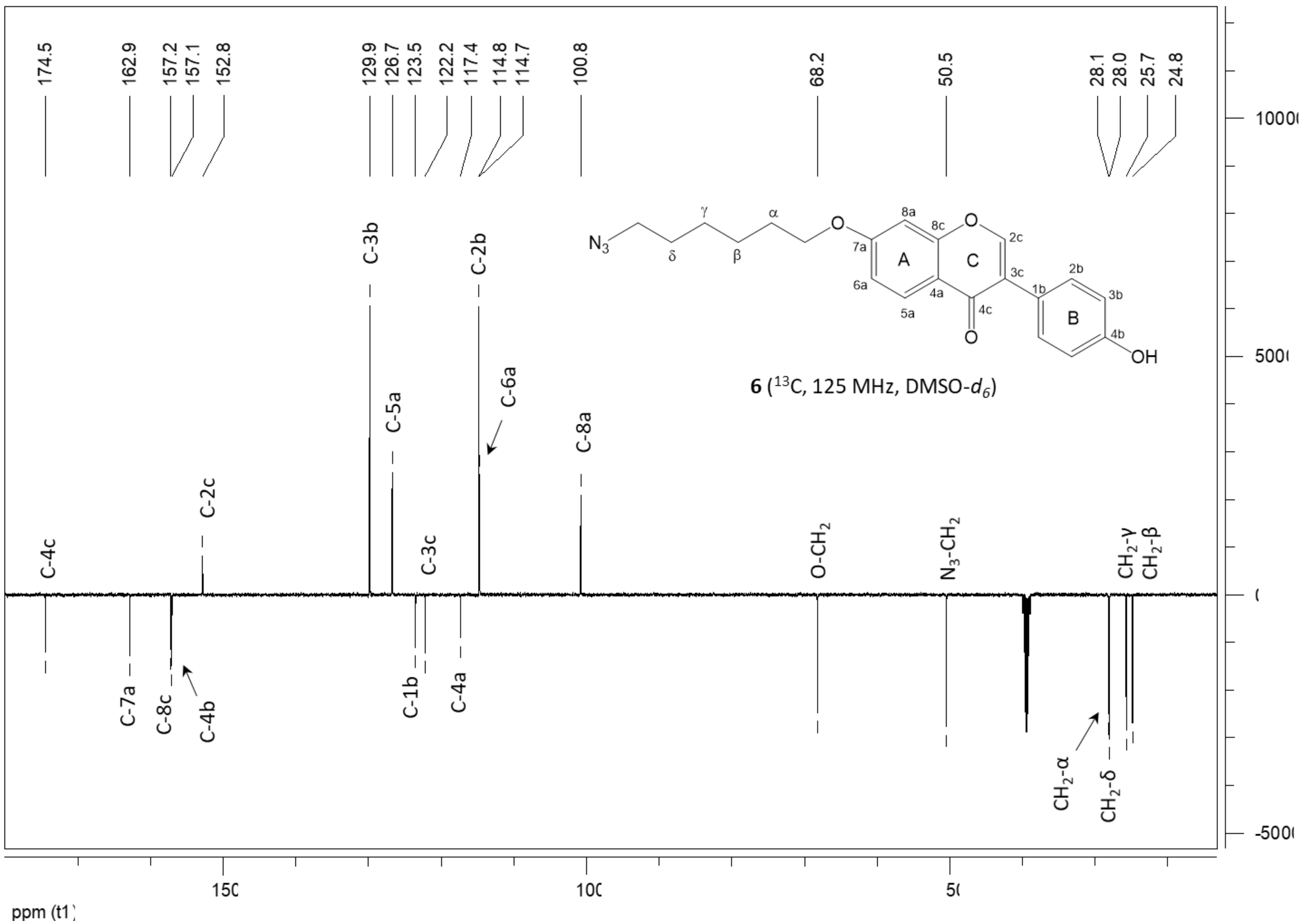

Figure S12. ^{13}C-apt NMR spectrum of 7-azidohexyl-daidzein (**6**)

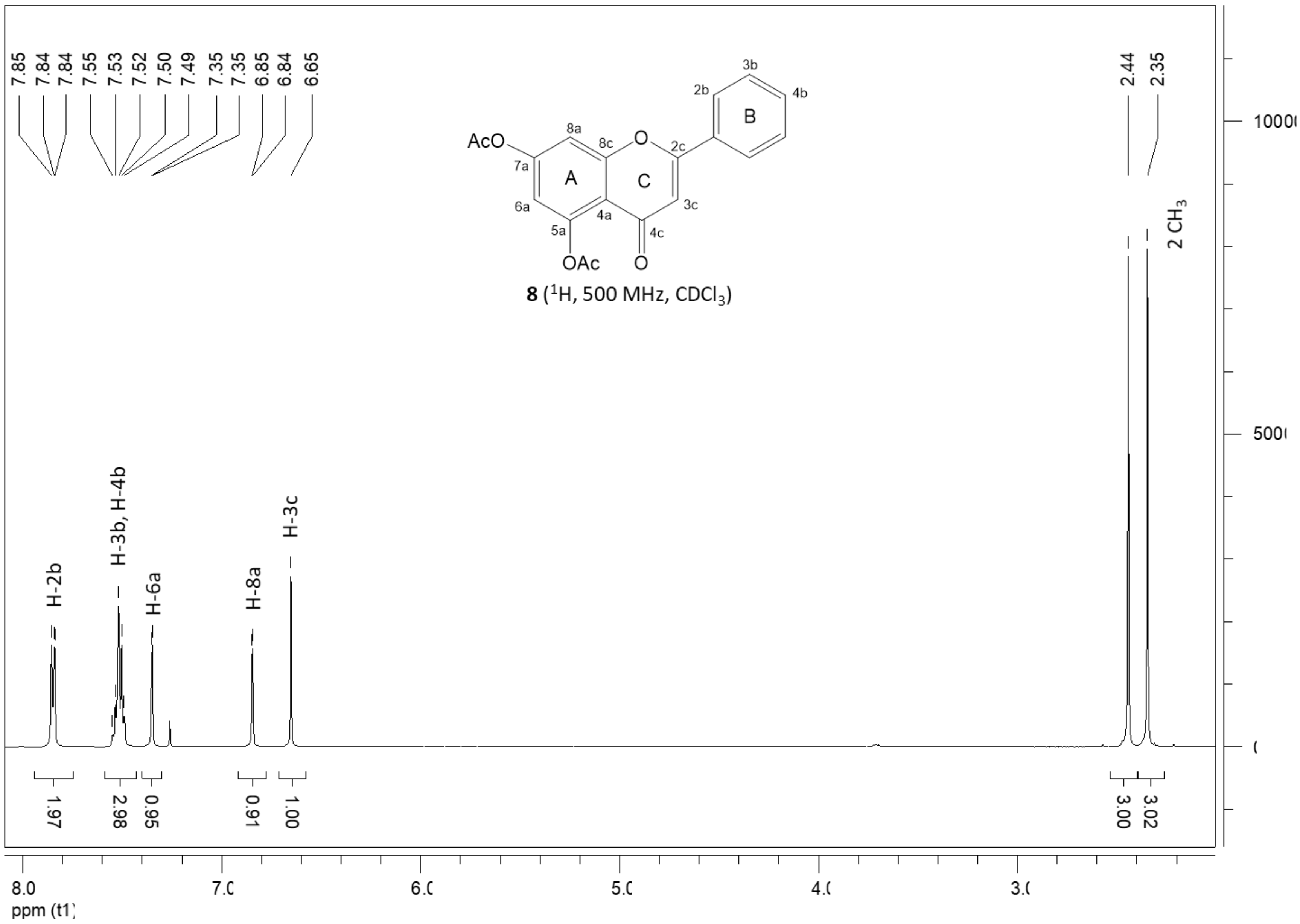

Figure S13. ^{1}H NMR spectrum of diacetyl chrysin (**8**)

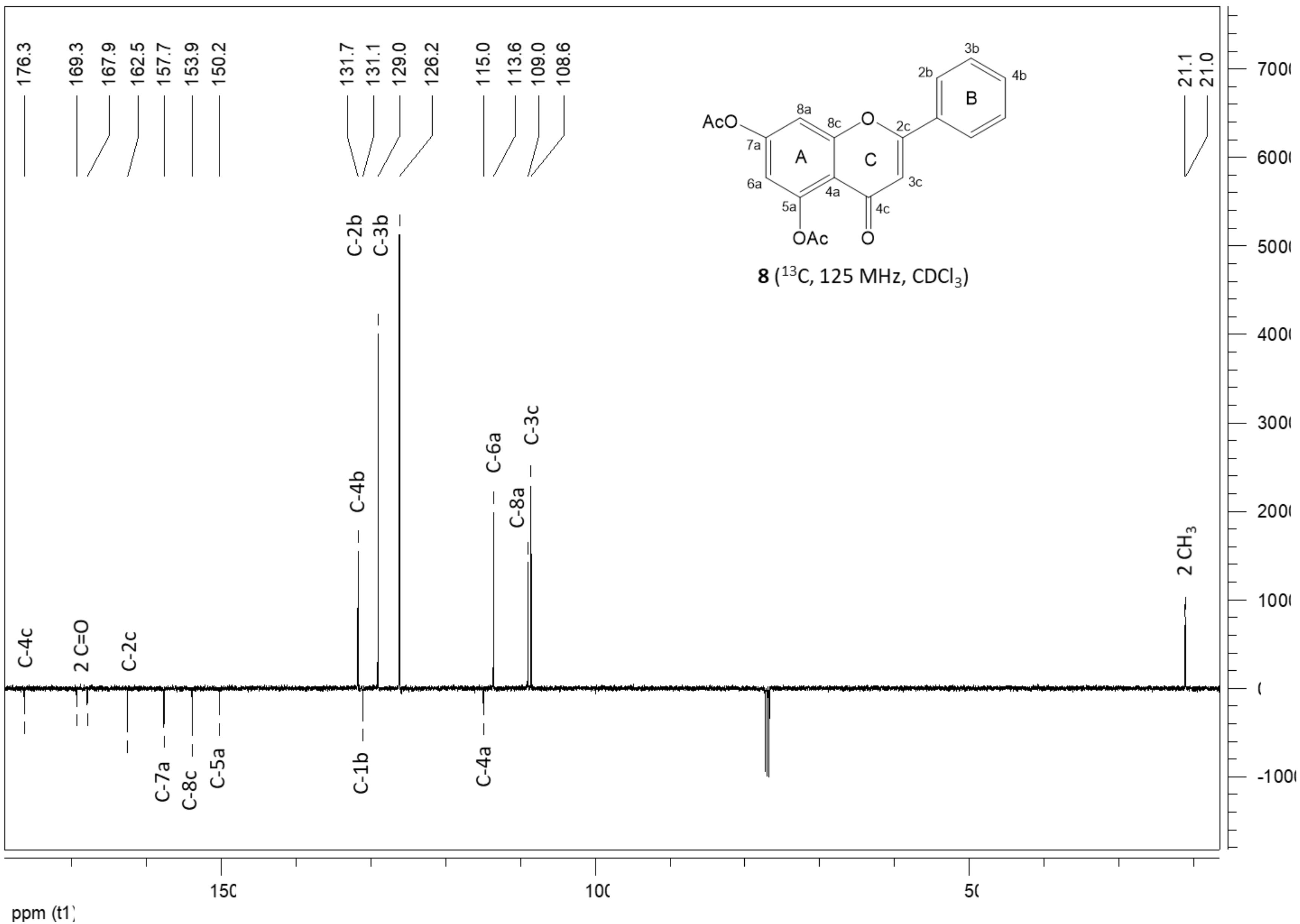

Figure S14. ^{13}C-apt NMR spectrum of diacetyl chrysin (**8**)

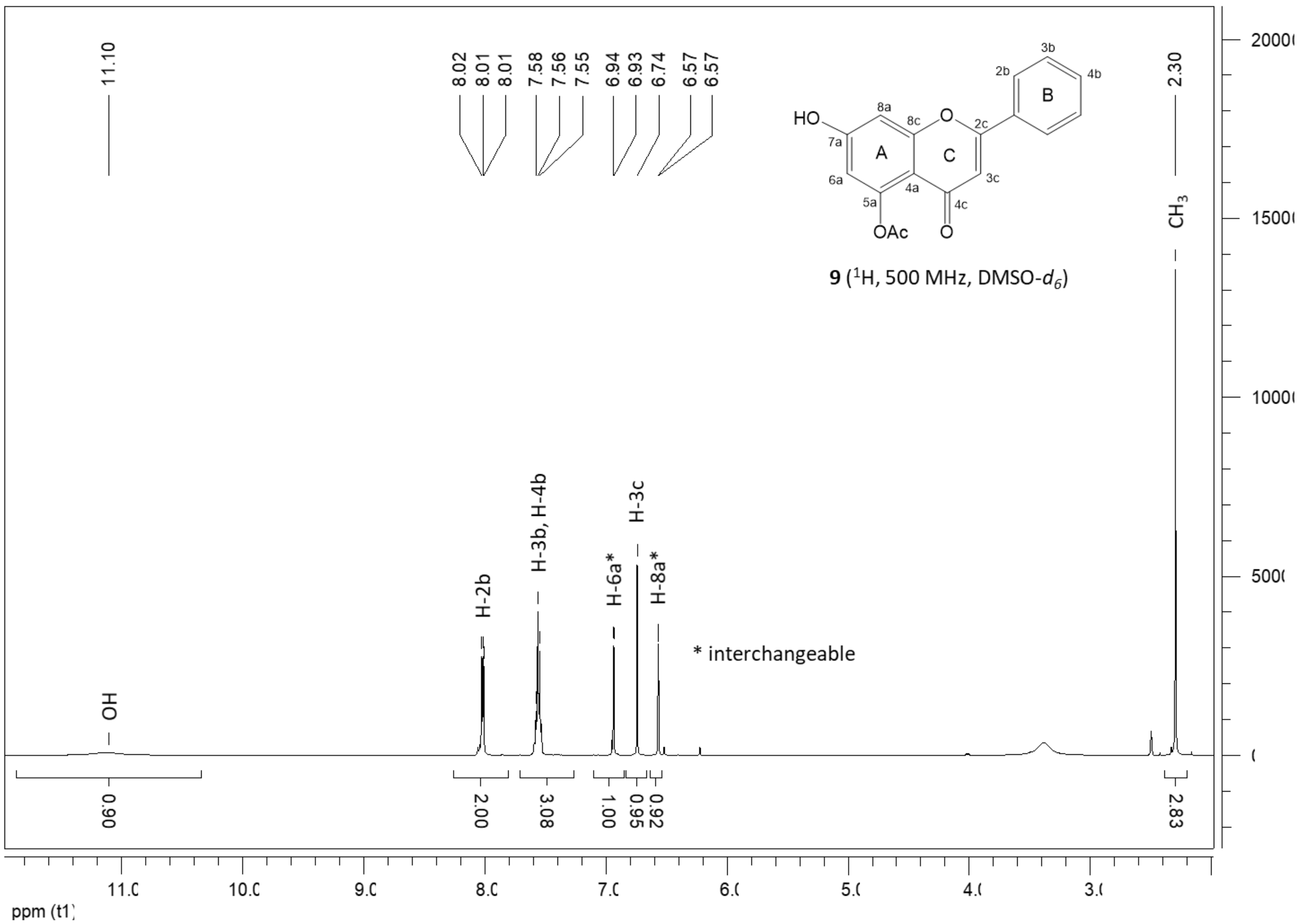

Figure S15. ^{1}H NMR spectrum of 5-acetylchrysin (**9**)

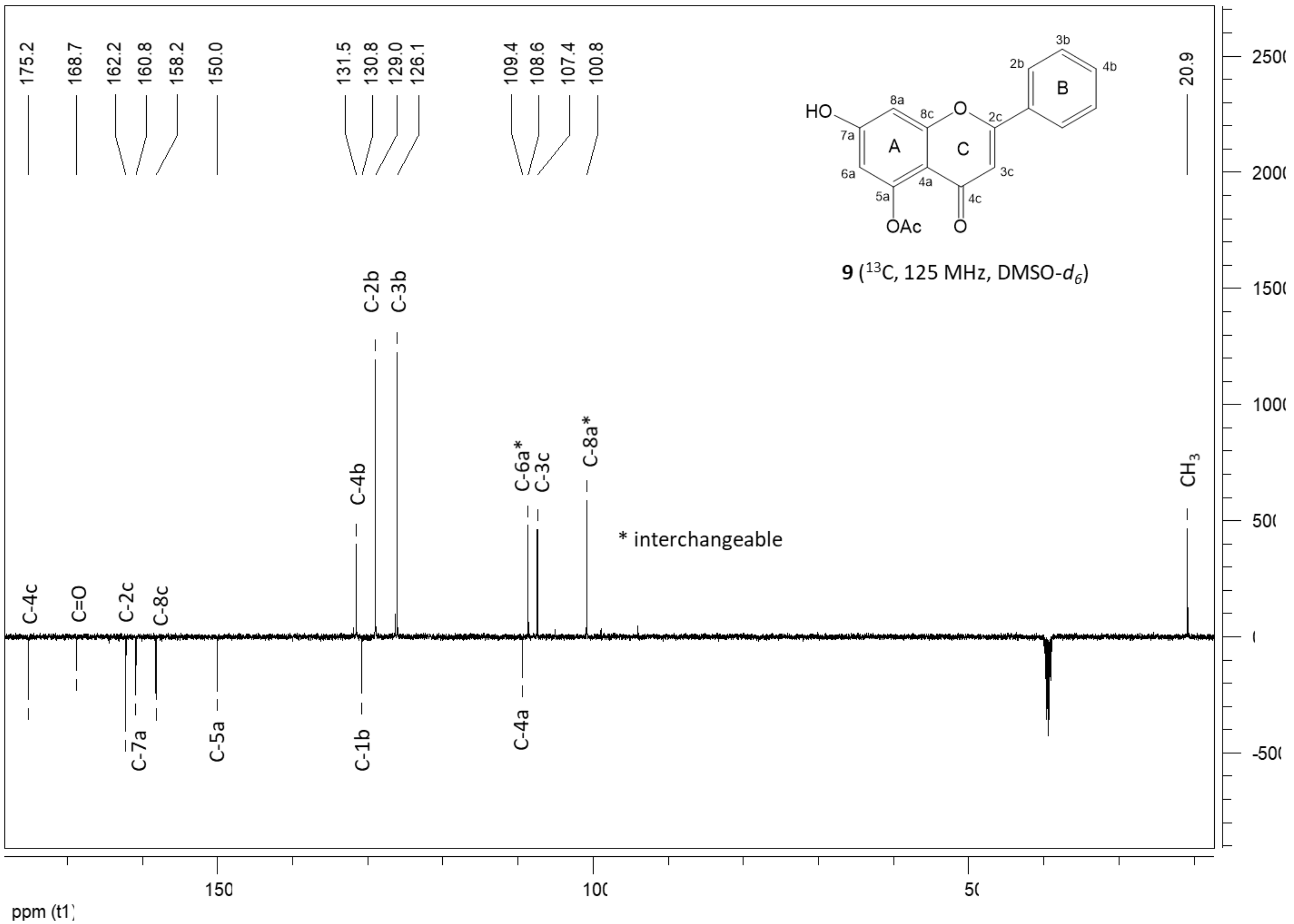

Figure S16. ^{13}C-apt NMR spectrum of 5-acetylchrysin (**9**)

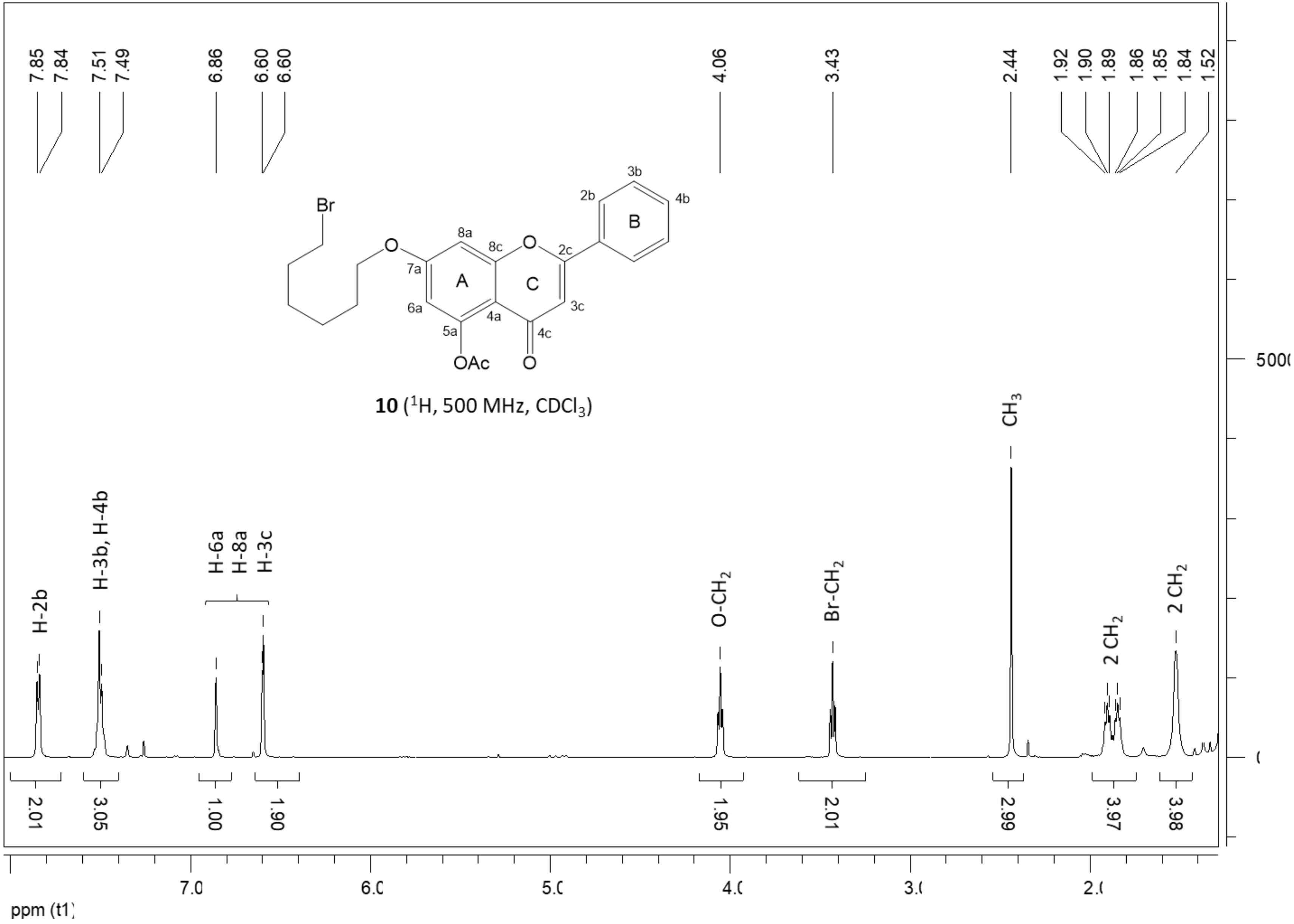

Figure S17. ^{1}H NMR spectrum of 7-bromohexyl-5-acetylchrysin (**10**)

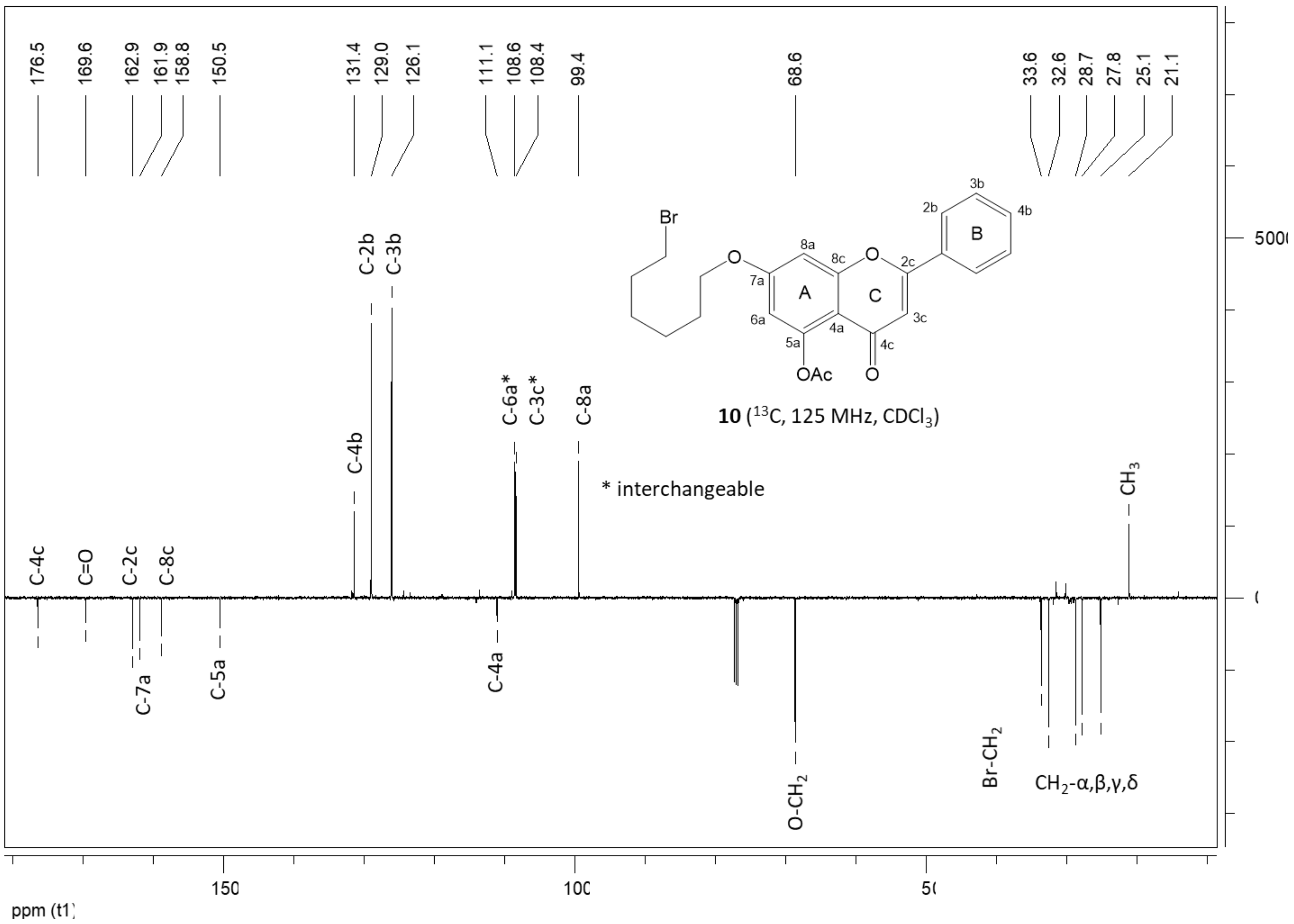

Figure S18. ^{13}C-apt NMR spectrum of 7-bromohexyl-5-acetylchrysin (**10**)

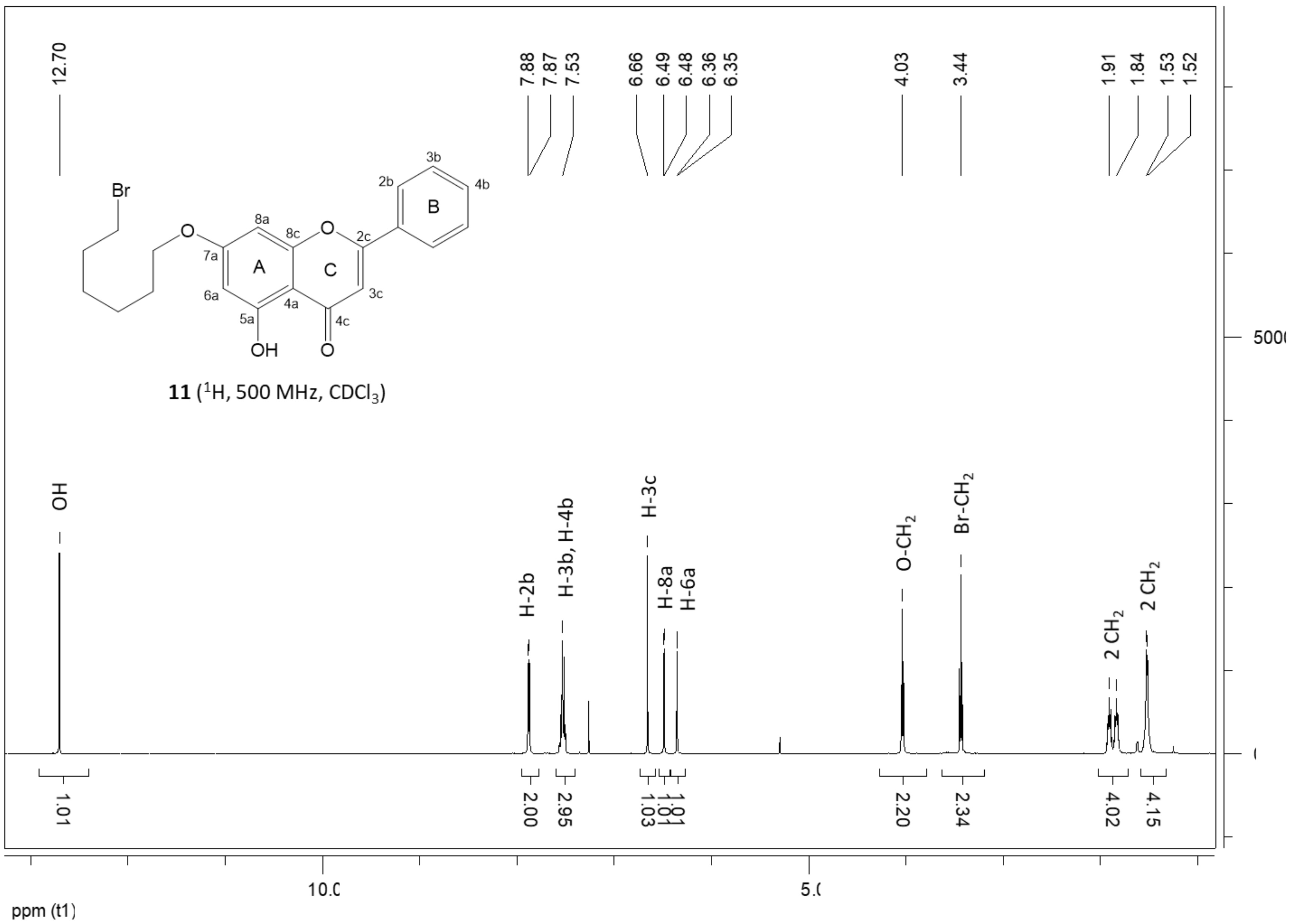

Figure S19. ^{1}H NMR spectrum of 7-bromohexyl-chrysin (**11**)

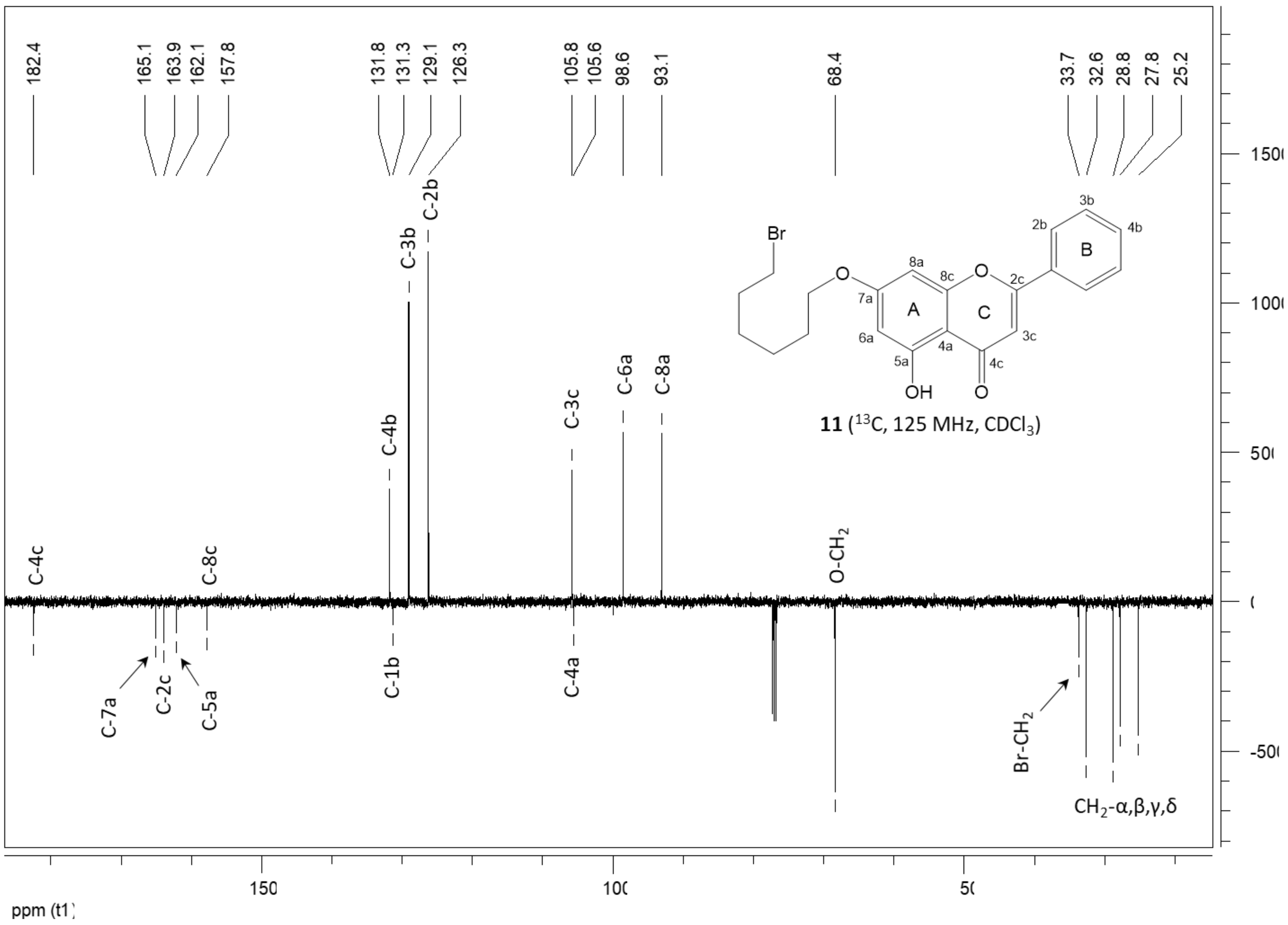

Figure S20. ^{13}C-apt NMR spectrum of 7-bromohexyl-chrysin (**11**)

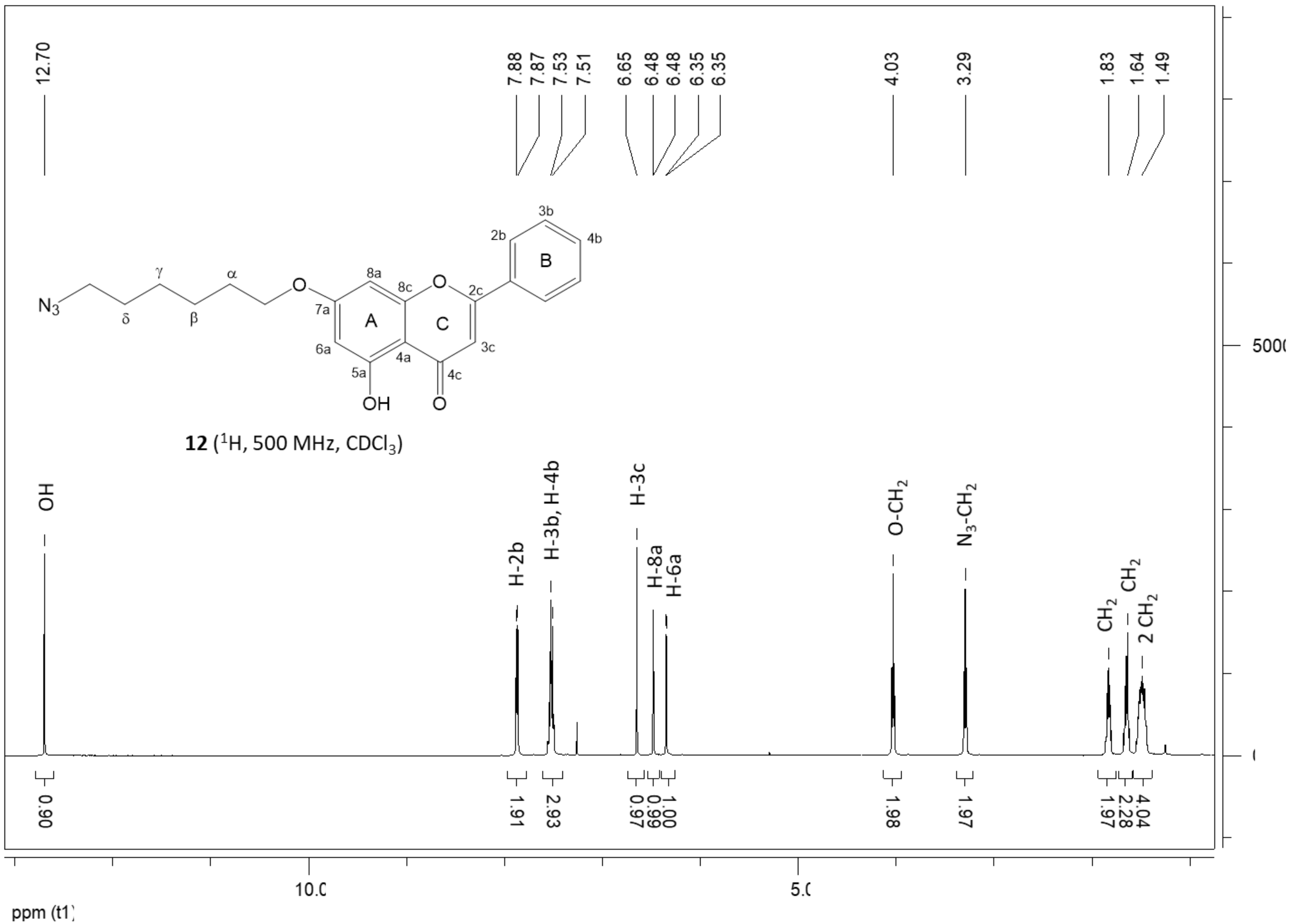

Figure S21. ^{1}H NMR spectrum of 7-azidohexyl-chrysin (**12**)

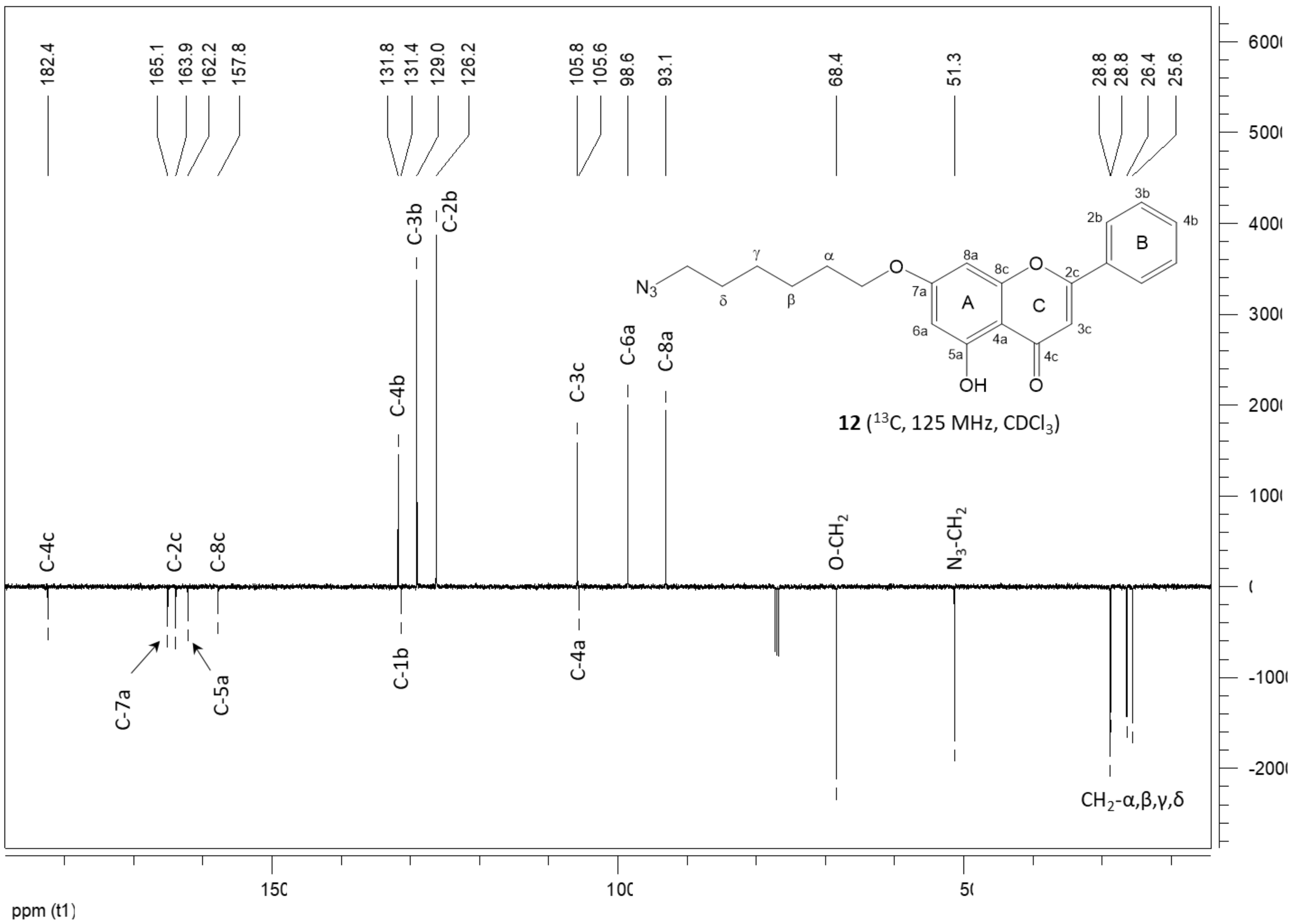

Figure S22. ^{13}C-apt NMR spectrum of 7-azidohexyl-chrysin (**12**)

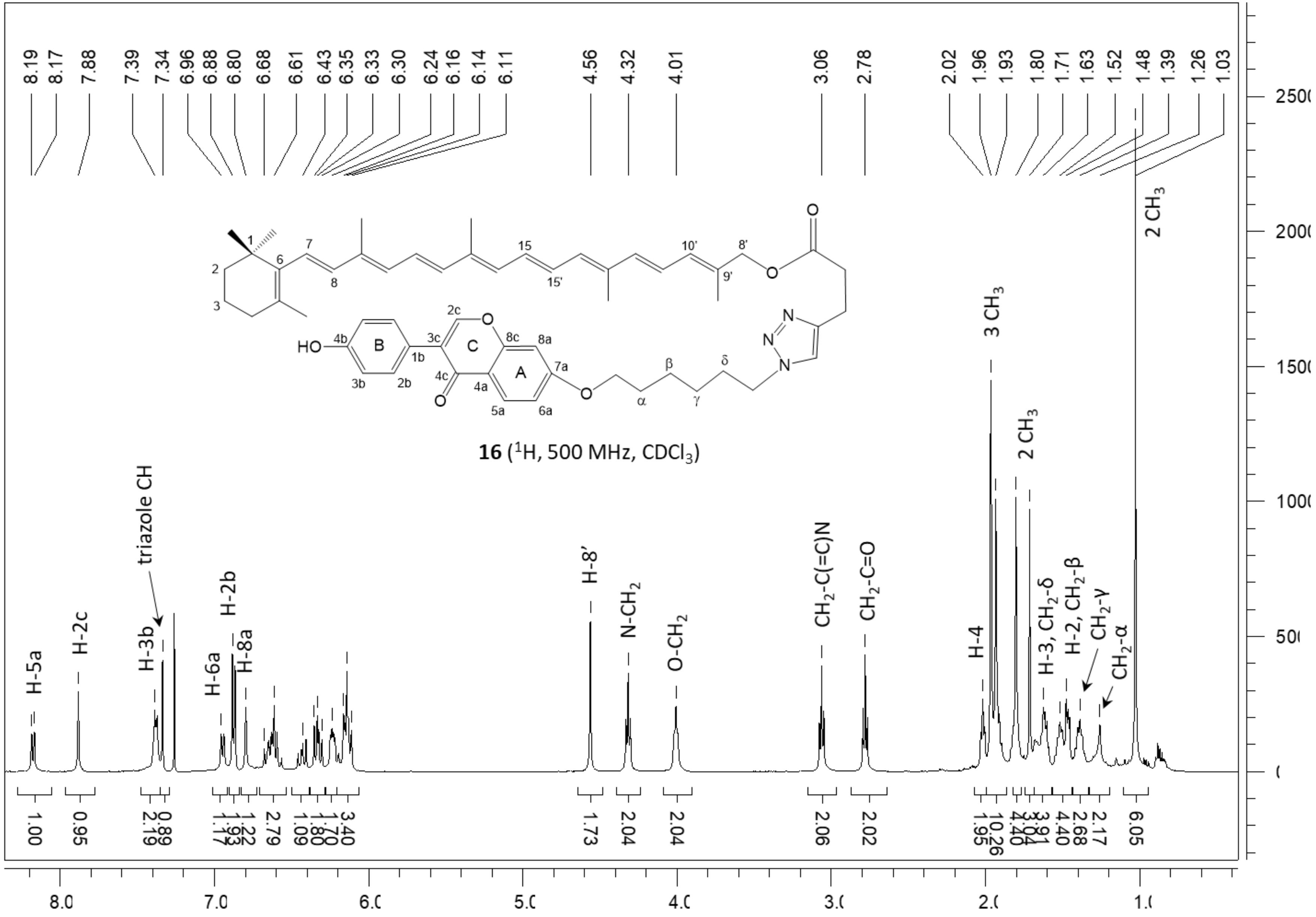

Figure S23.a. ^{1}H NMR spectrum of daidzein-8′-apo-β-carotenol conjugate (**16**)

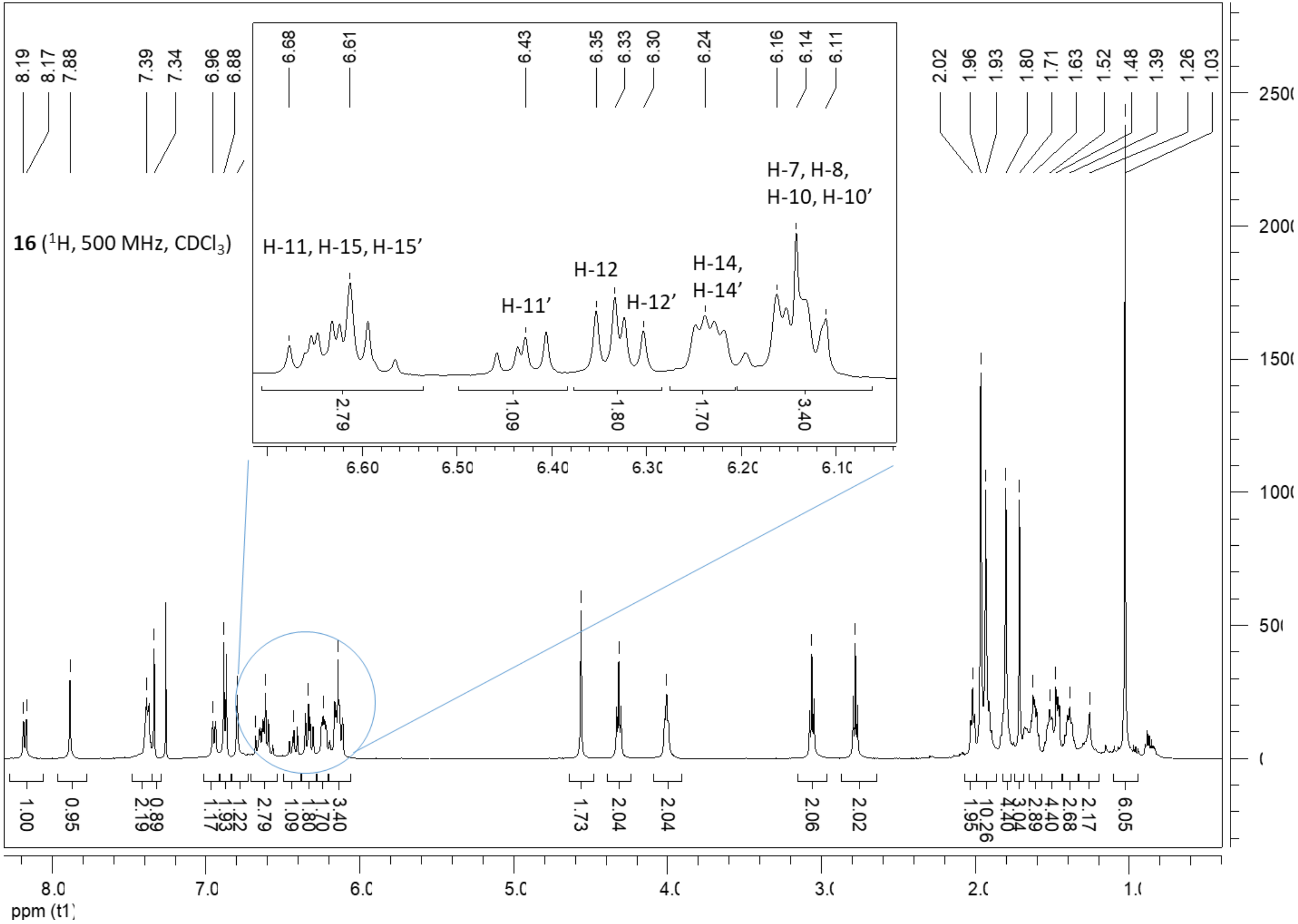

Figure S23.b. ^{1}H NMR spectrum of daidzein-8′-apo-β-carotenol conjugate (**16**)

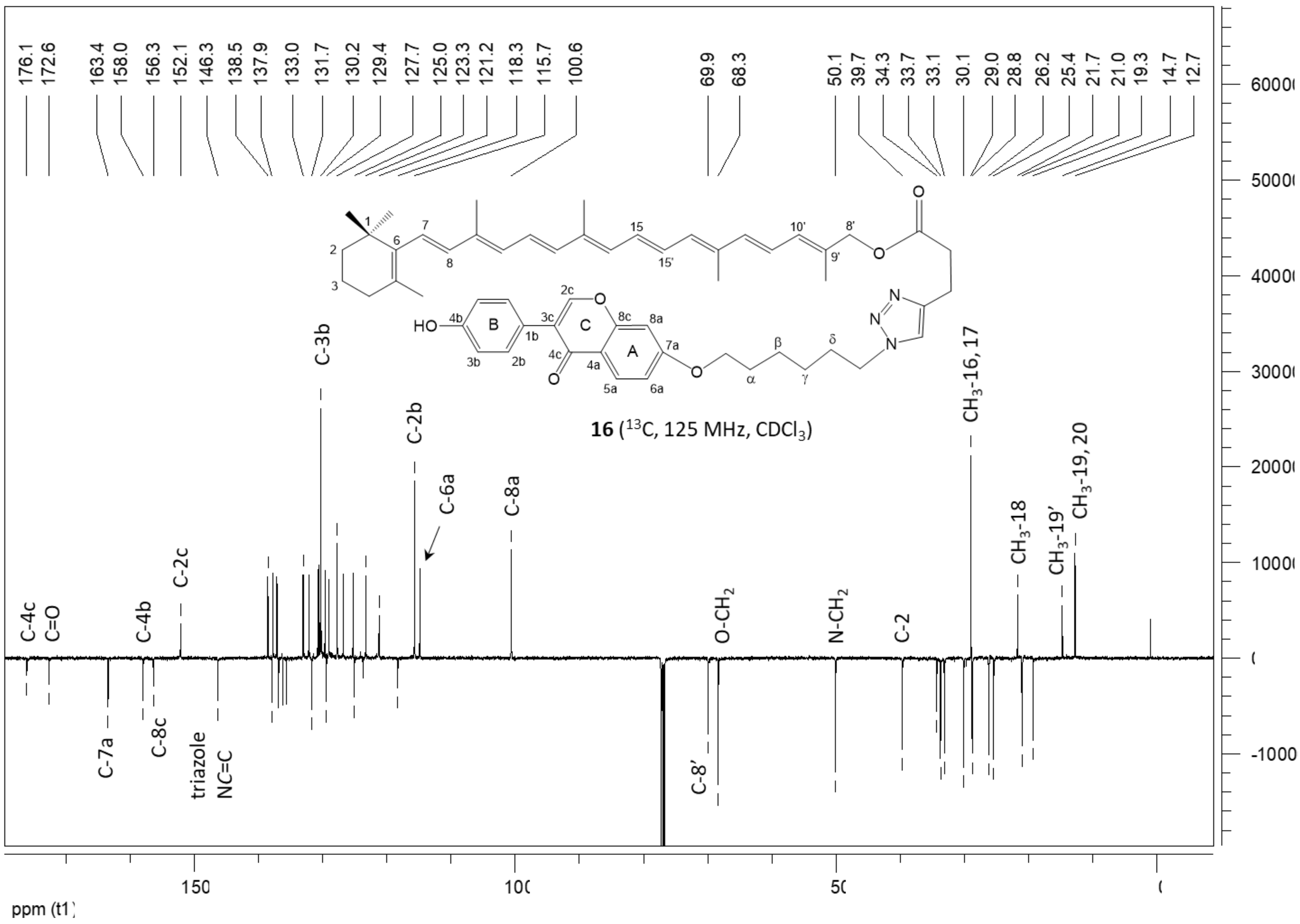

Figure S24.a. ^{13}C-apt NMR spectrum of daidzein-8′-apo-β-carotenol conjugate (**16**)

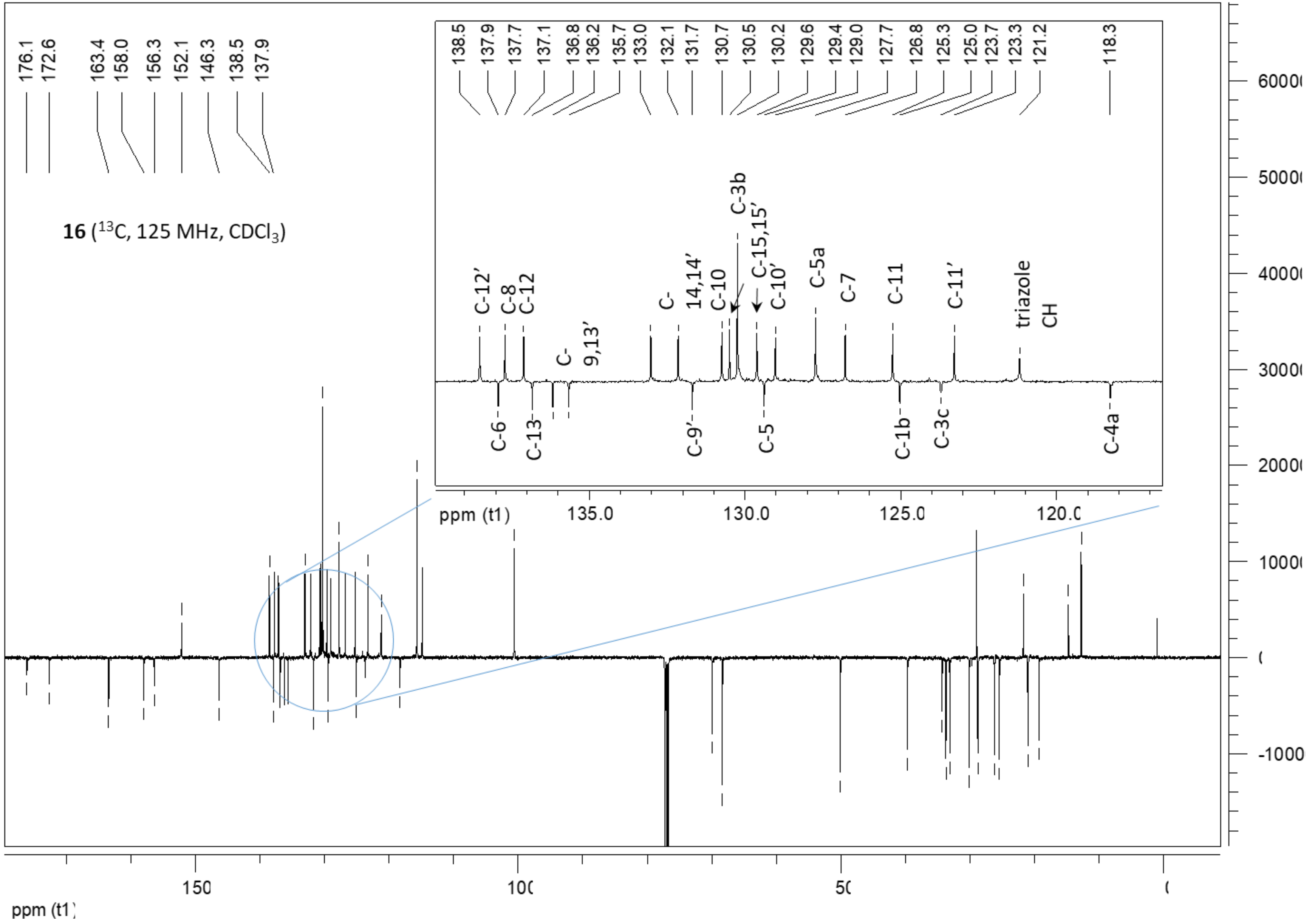

Figure S24.b. ^{13}C-apt NMR spectrum of daidzein-8′-apo-β-carotenol conjugate (**16**)

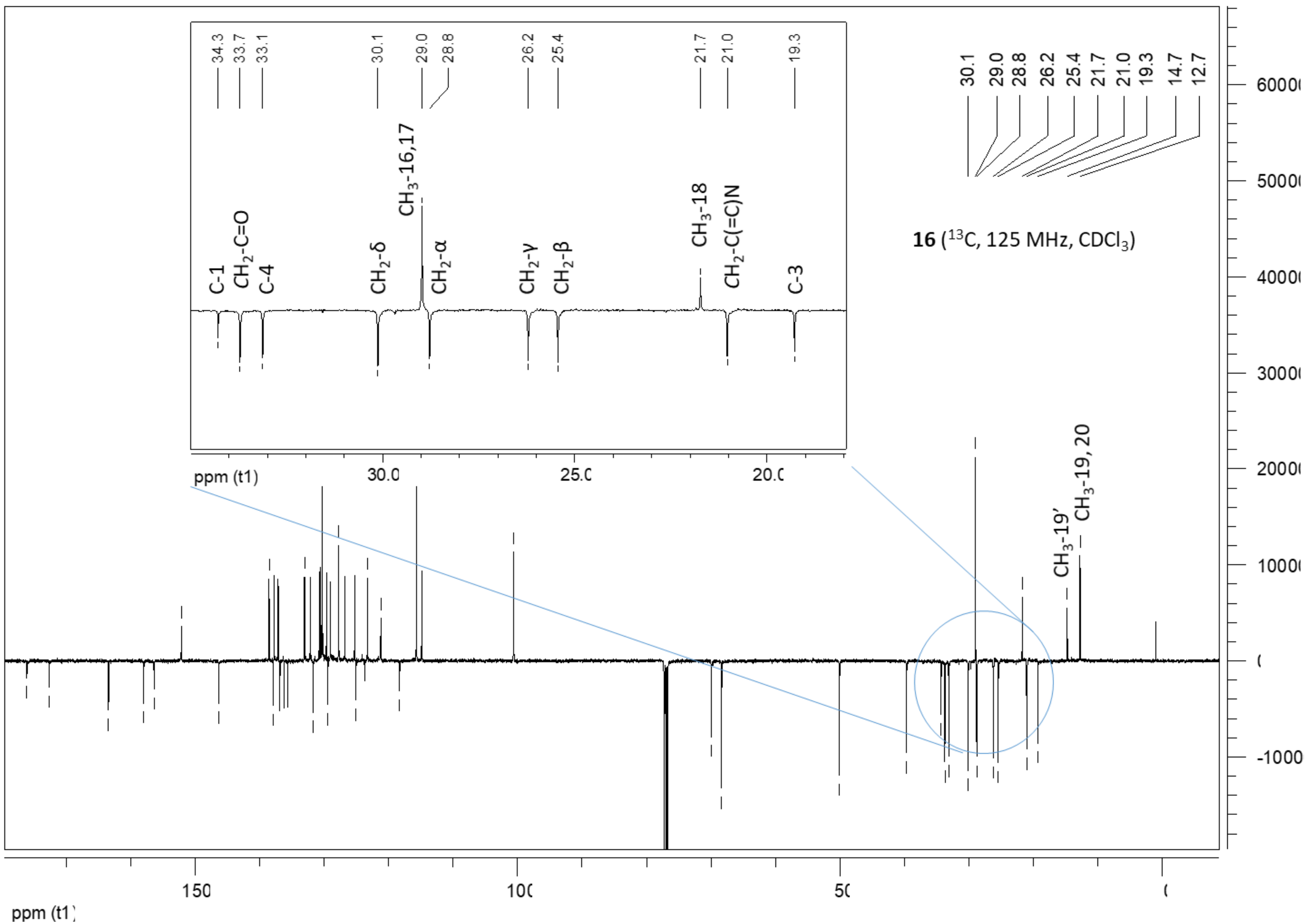

Figure S24.c. ^{13}C-apt NMR spectrum of daidzein-8′-apo-β-carotenol conjugate (**16**)

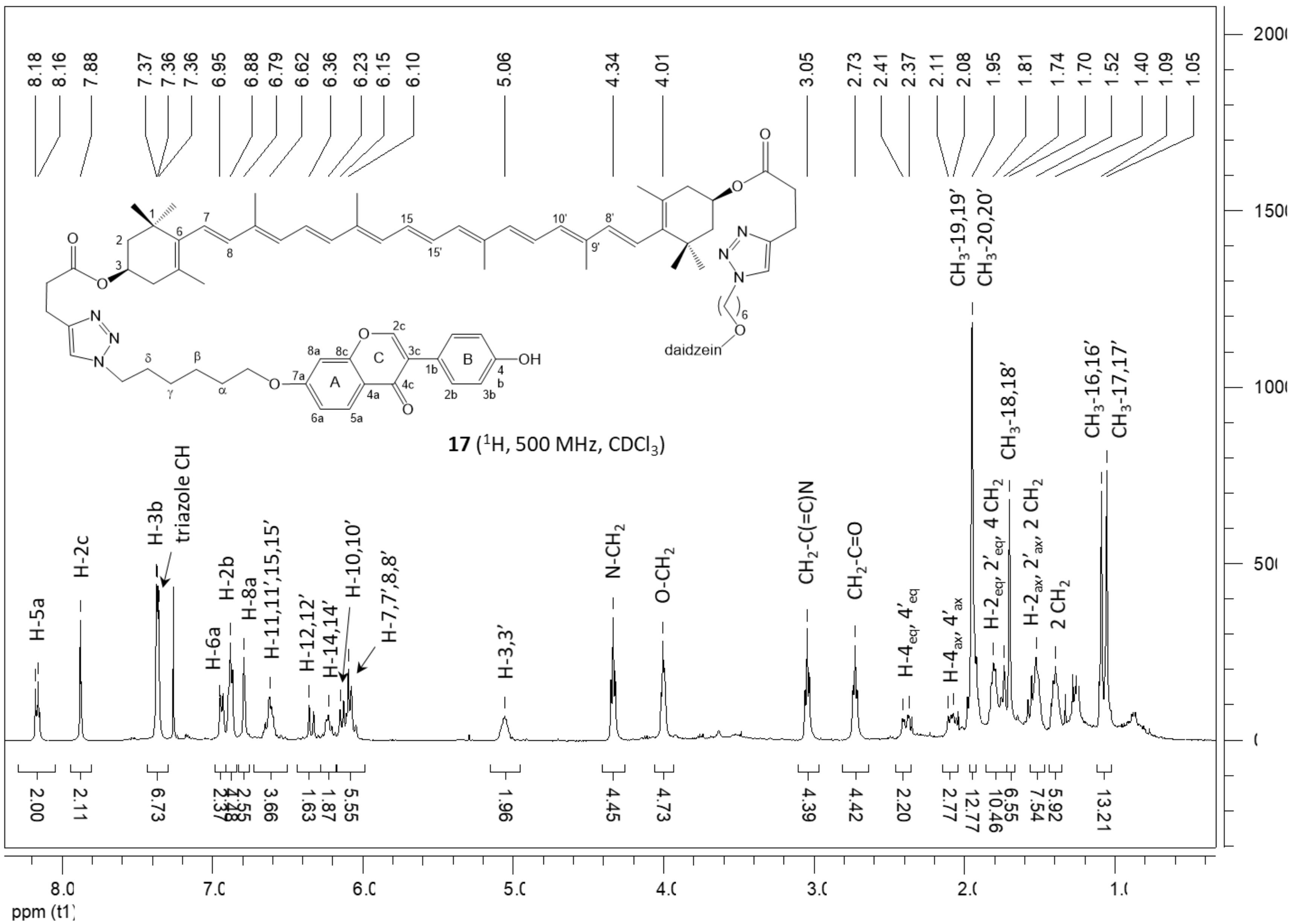

Figure S25. ^{1}H NMR spectrum of *bis*-daidzein-zeaxanthin conjugate (**17**)

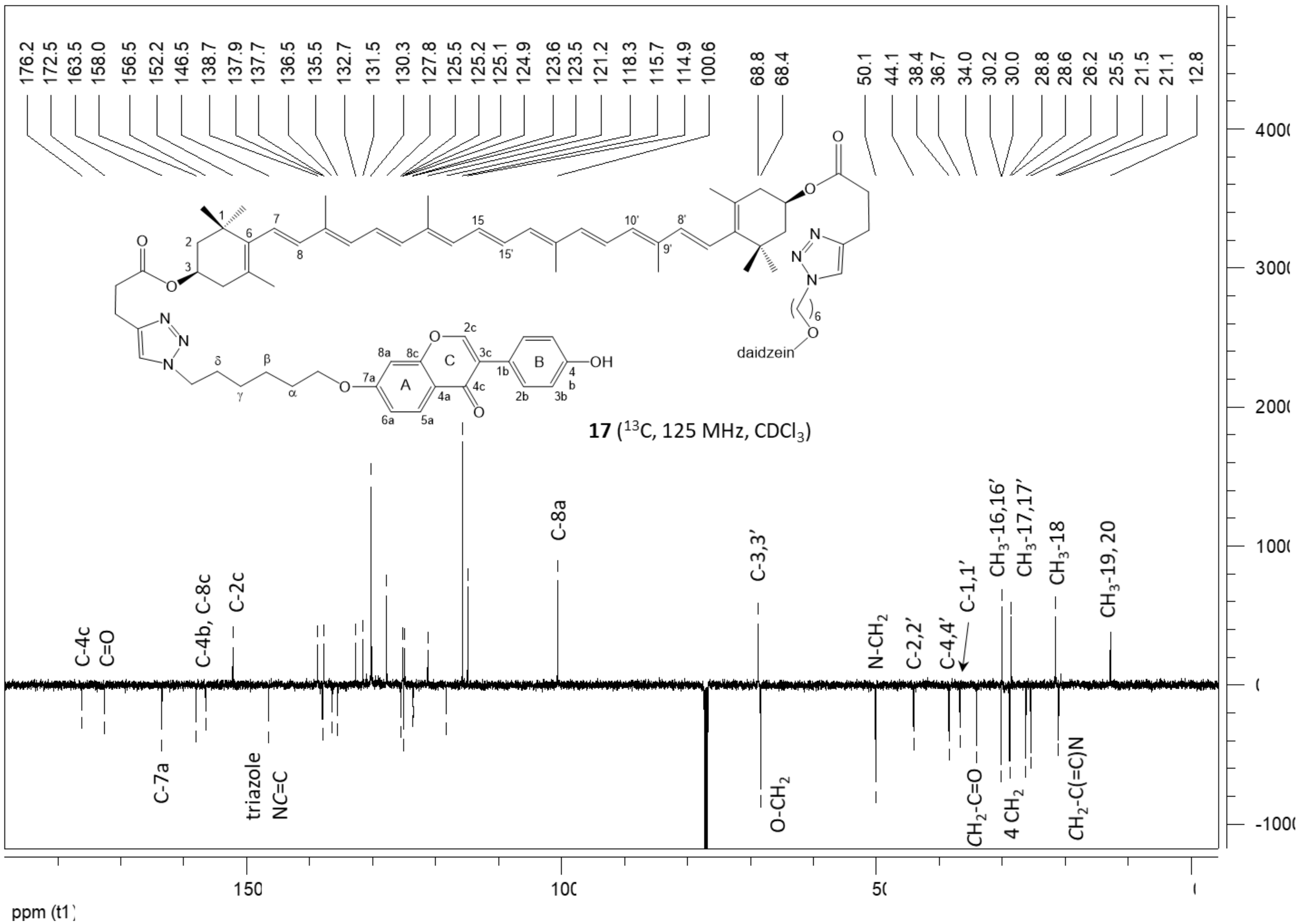

Figure S26.a. ^{13}C-apt NMR spectrum of *bis*-daidzein-zeaxanthin conjugate (**17**)

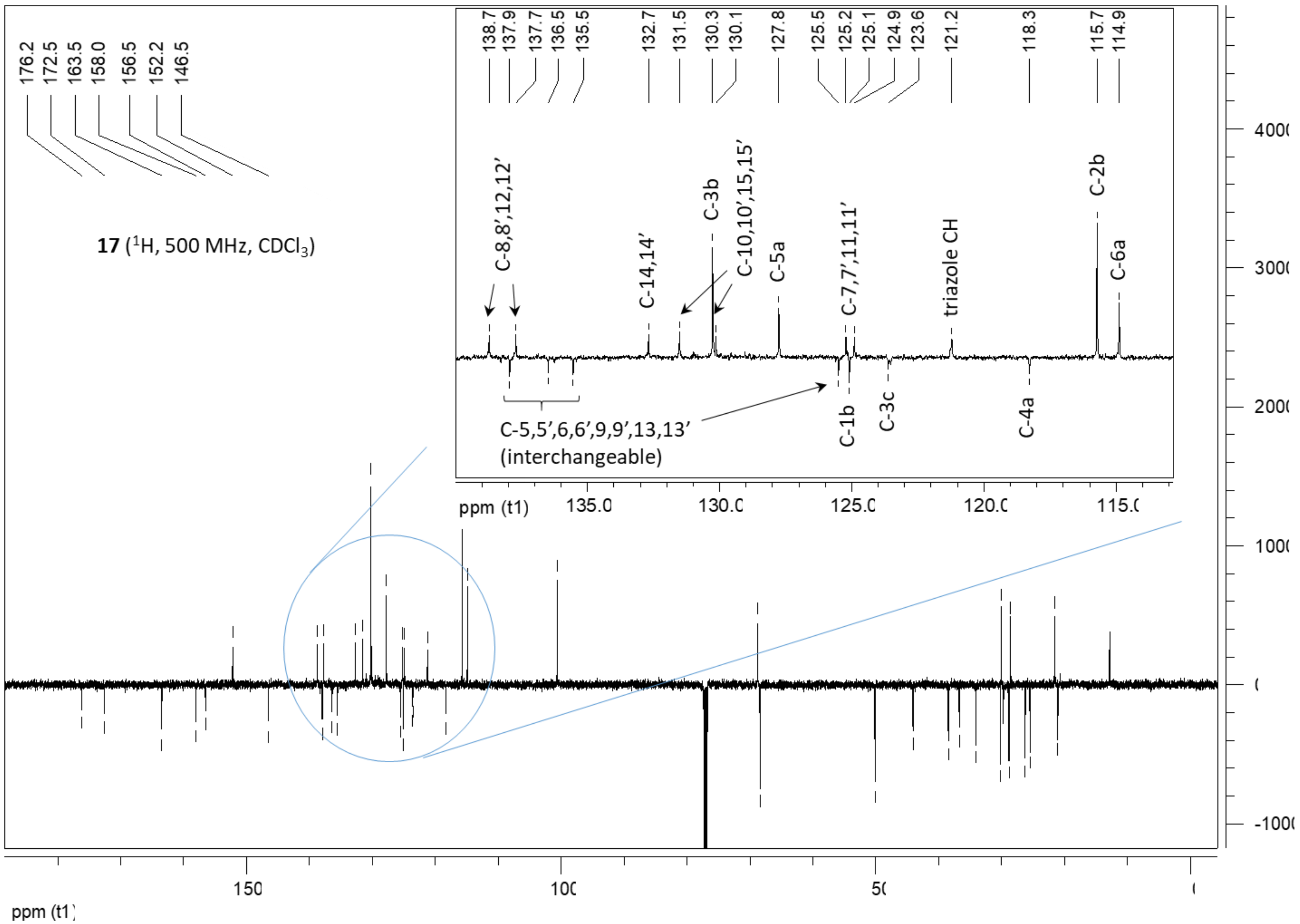

Figure S26.b. ^{13}C-apt NMR spectrum of *bis*-daidzein-zeaxanthin conjugate (**17**)

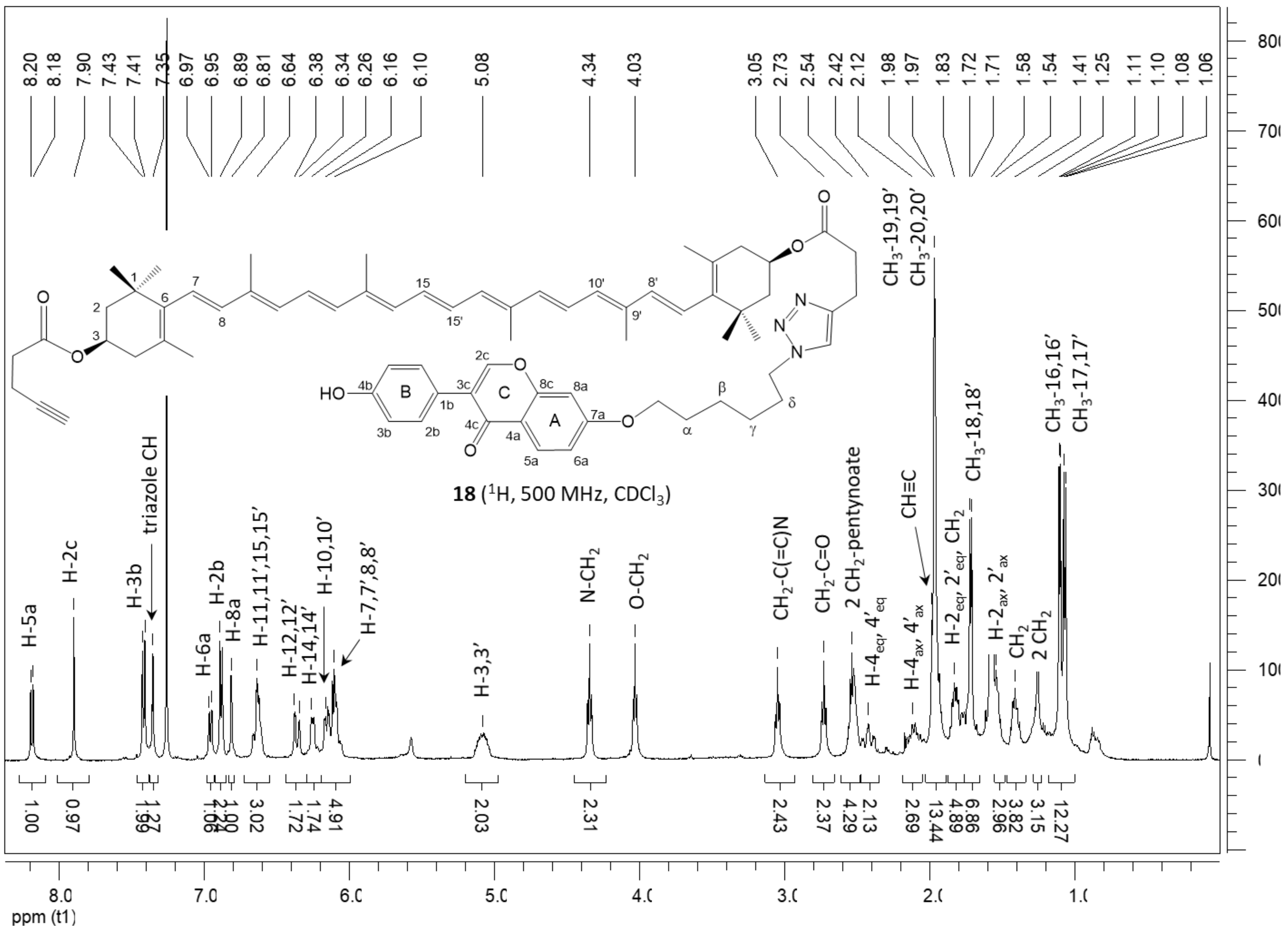

Figure S27. ^{1}H NMR spectrum of daidzein-zeaxanthin conjugate (**18**)

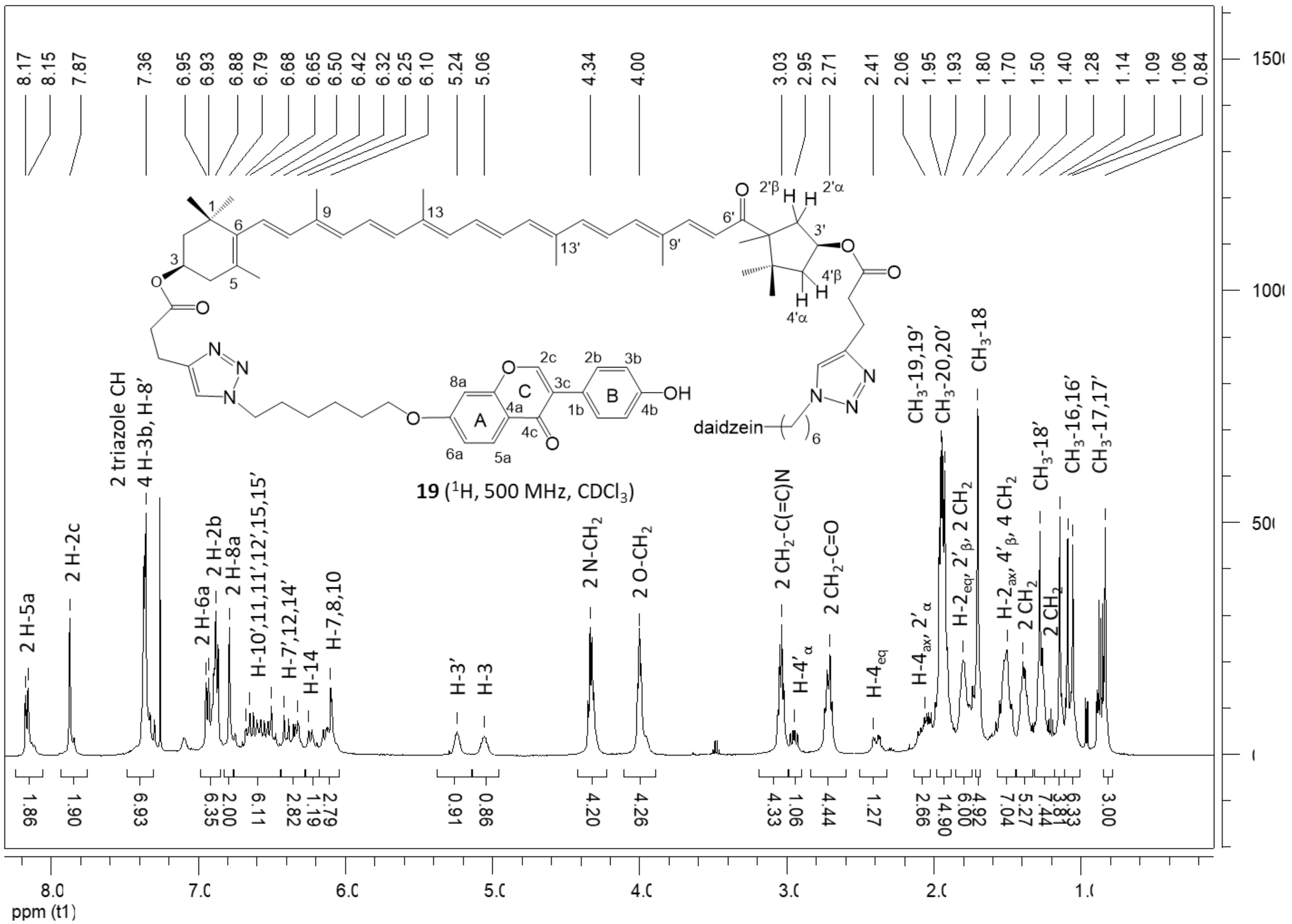

Figure S28. ^{1}H NMR spectrum of *bis*-daidzein-capsanthin conjugate (**19**)

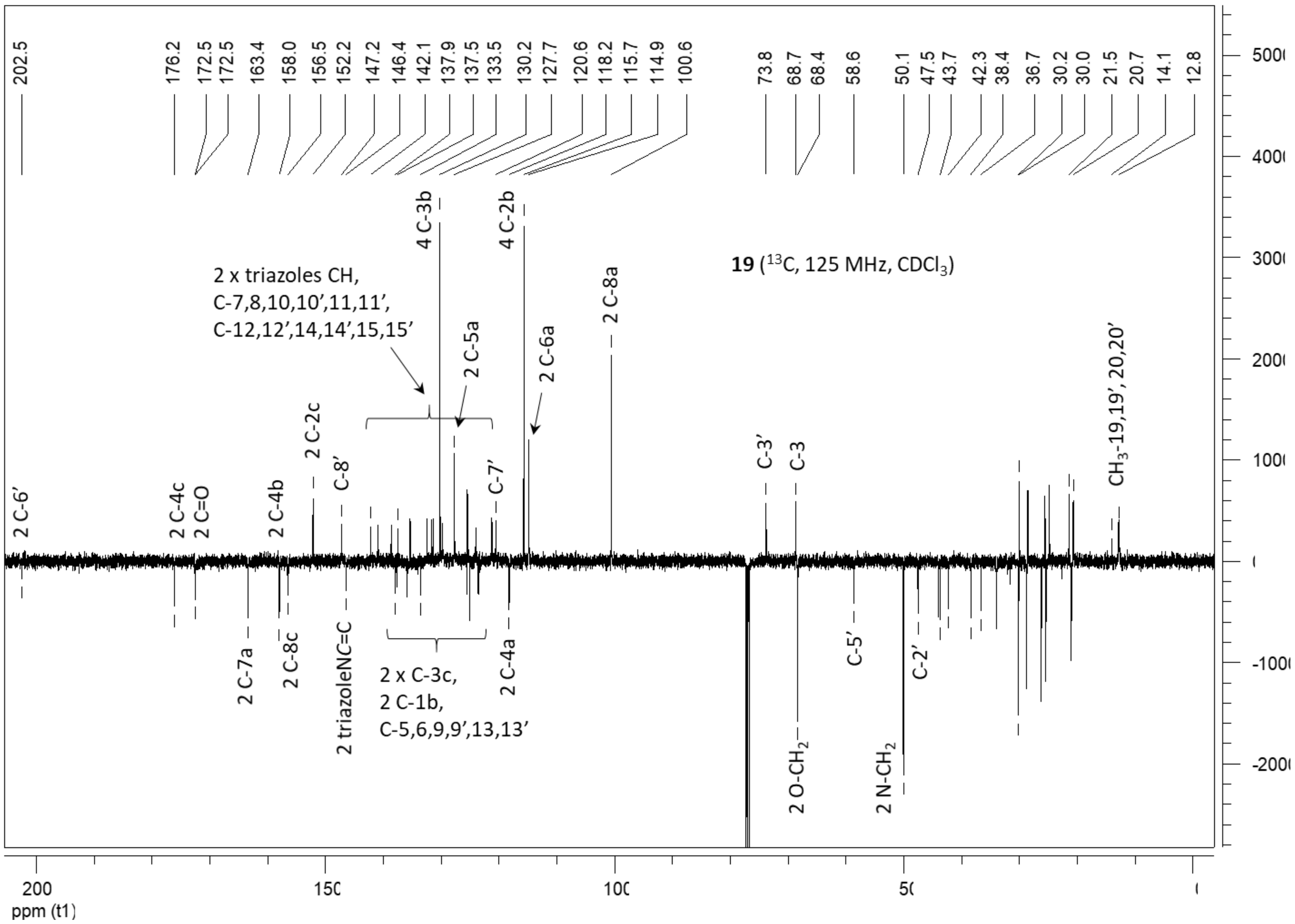

Figure S29.a. ^{13}C-apt NMR spectrum of *bis*-daidzein-capsanthin conjugate (**19**)

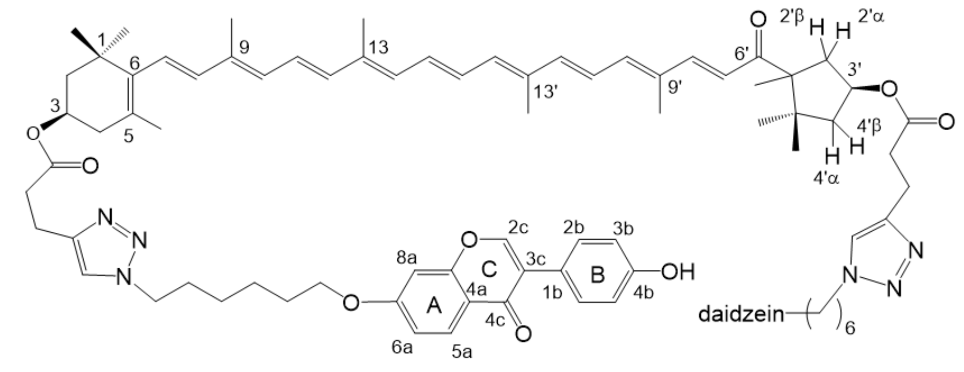

19 (^{13}C, 125 MHz, $CDCl_3$)

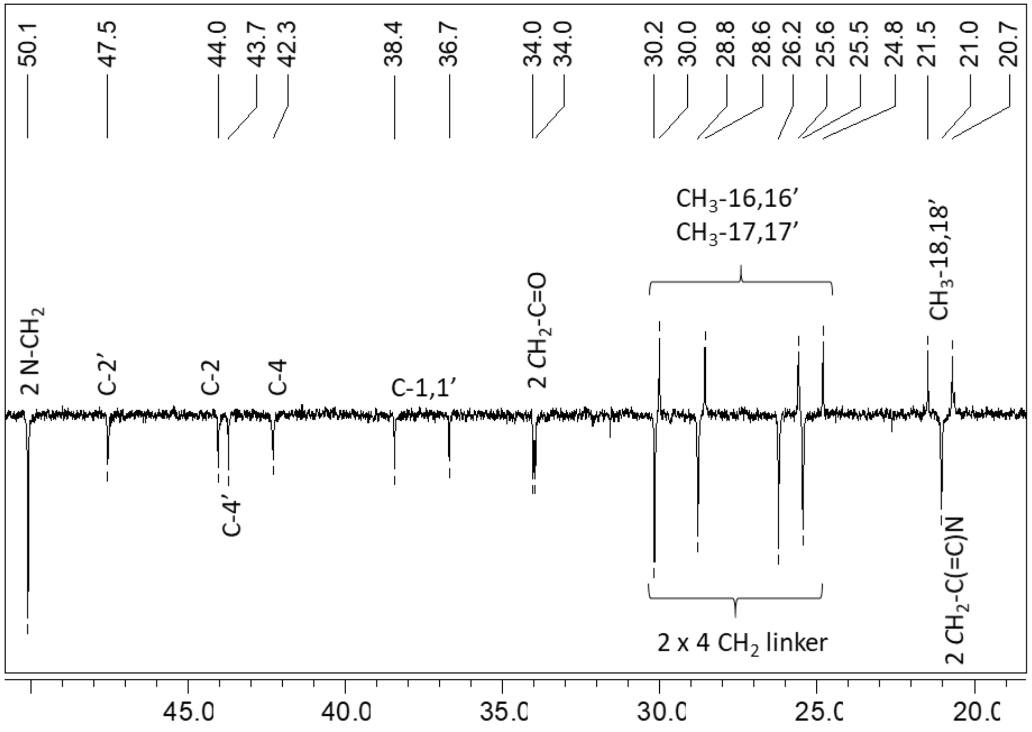

Figure S29.b. ^{13}C-apt NMR spectrum of *bis*-daidzein-capsanthin conjugate (**19**)

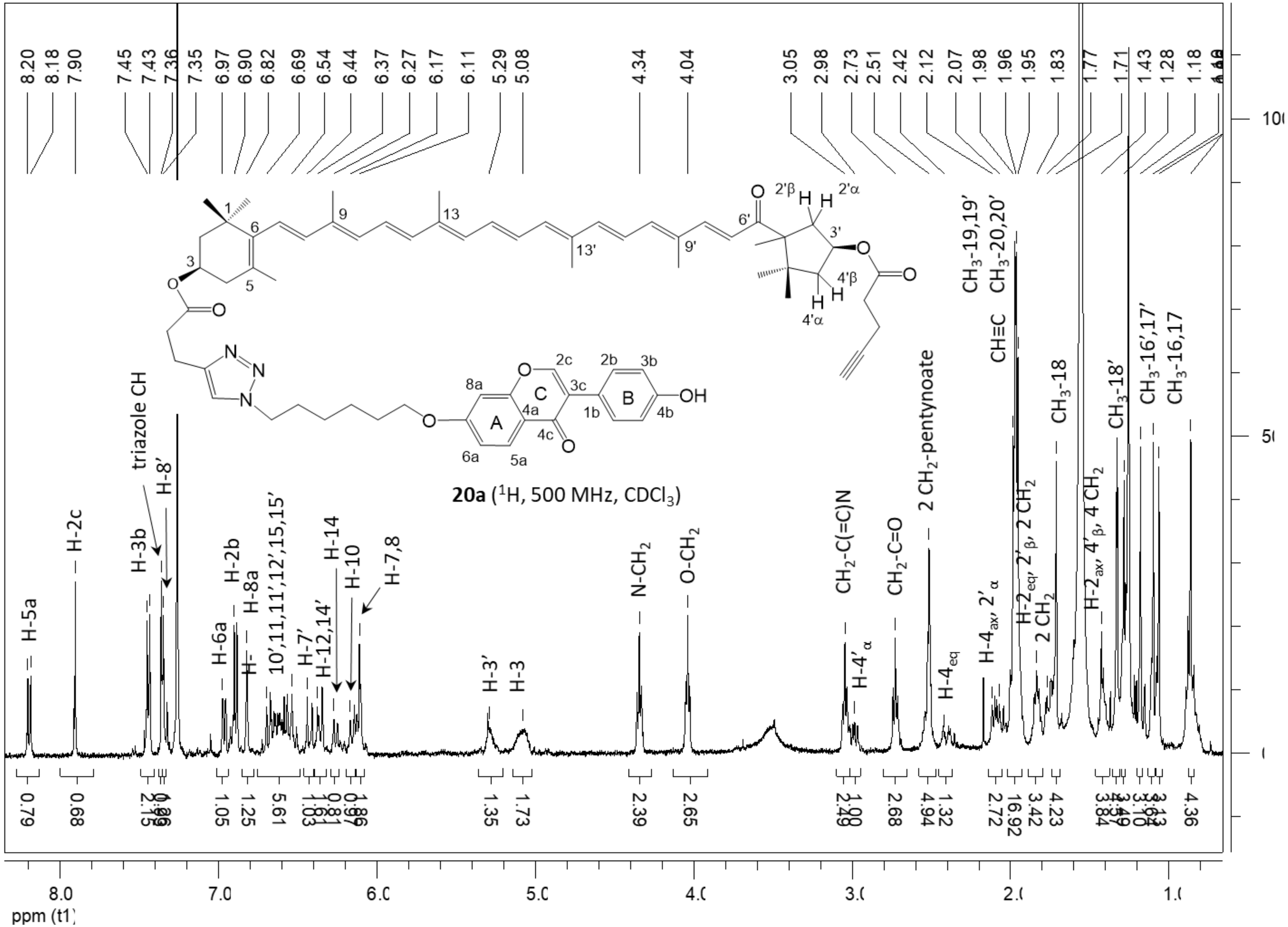

Figure S30. ^{1}H NMR spectrum of daidzein-capsanthin conjugate (**20a**)

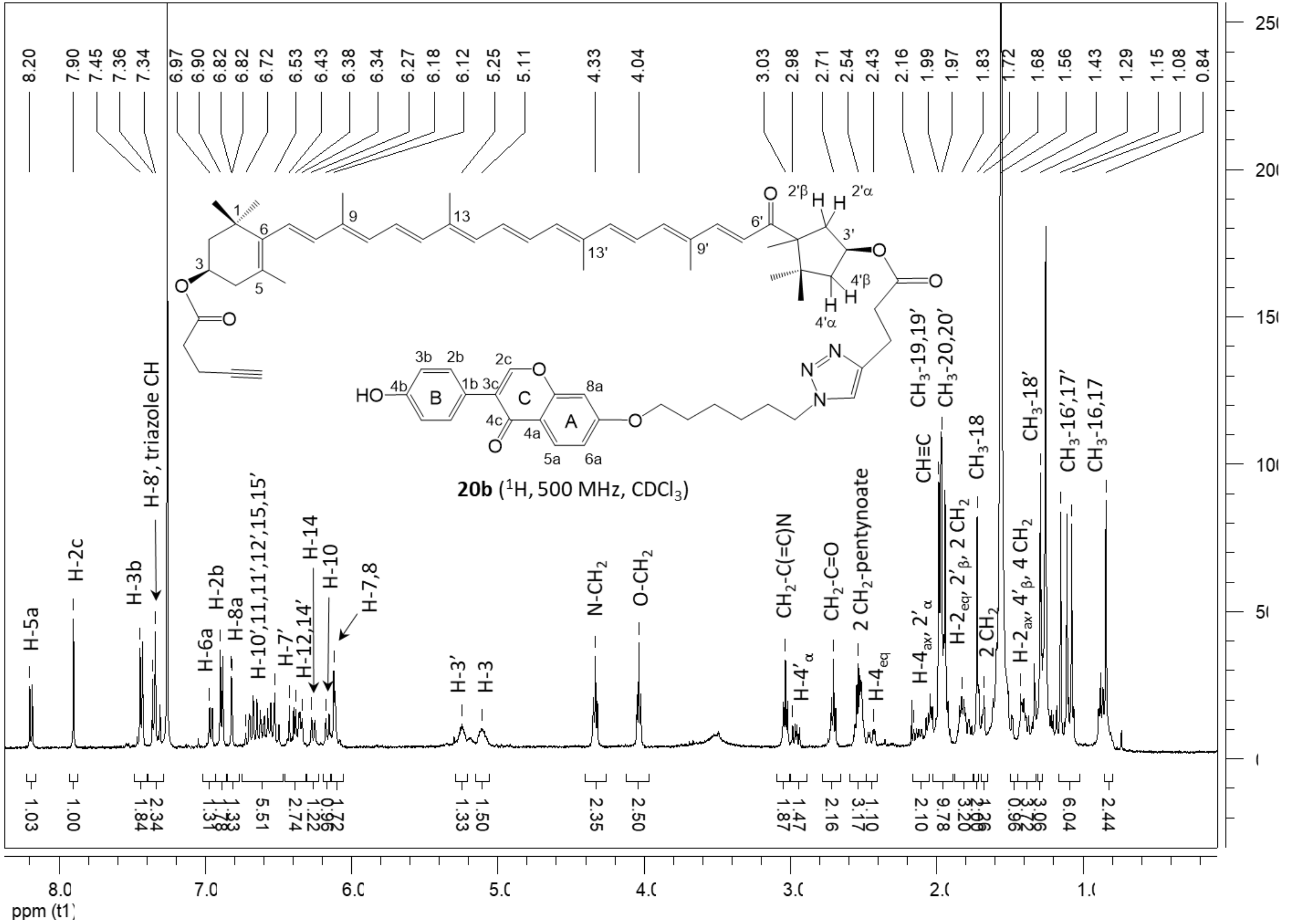

Figure S31. ^{1}H NMR spectrum of daidzein-capsanthin conjugate (**20b**)

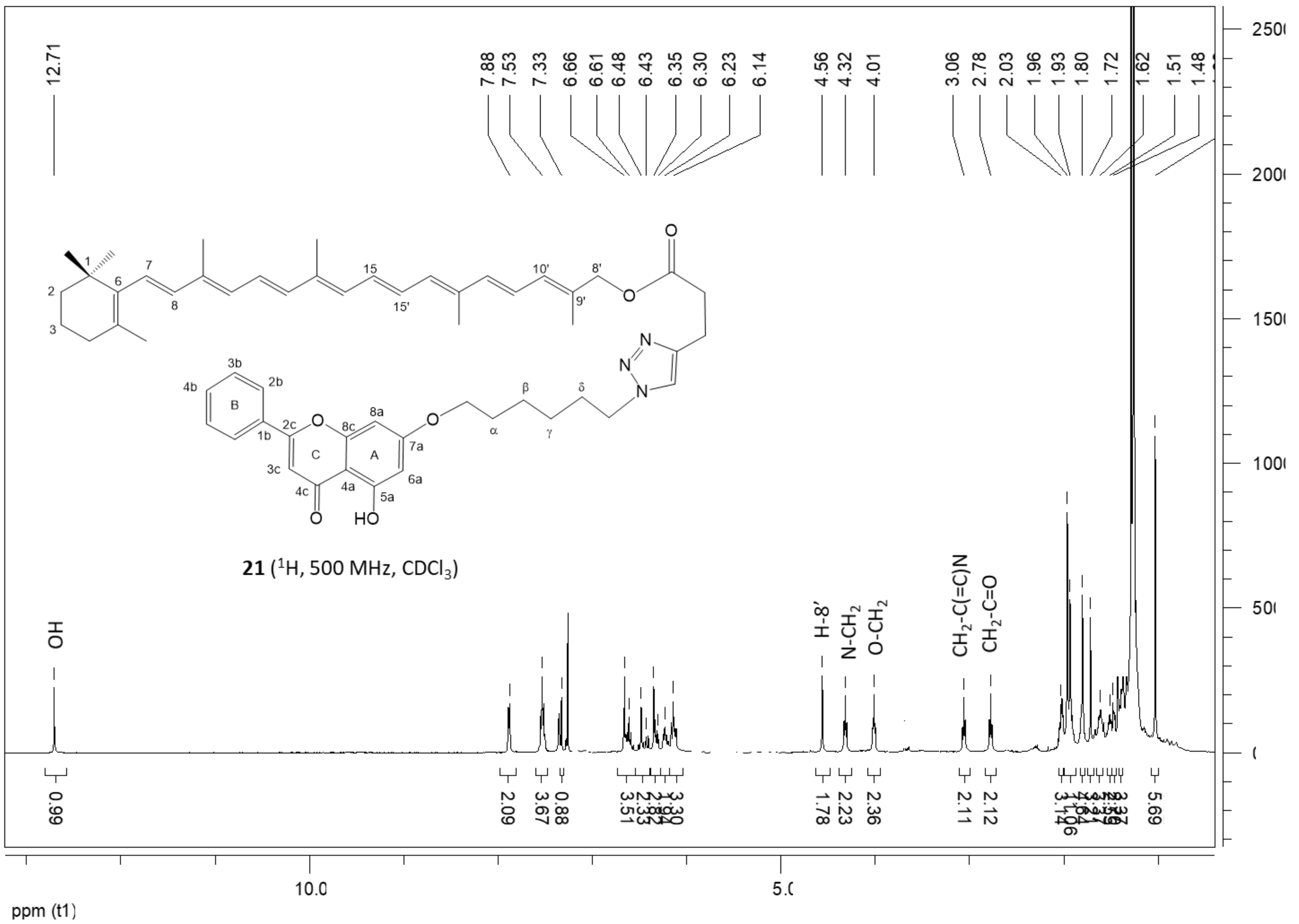

Figure S32.a. ¹H NMR spectrum of chrysin-8′-apo-β-carotenol conjugate (**21**)

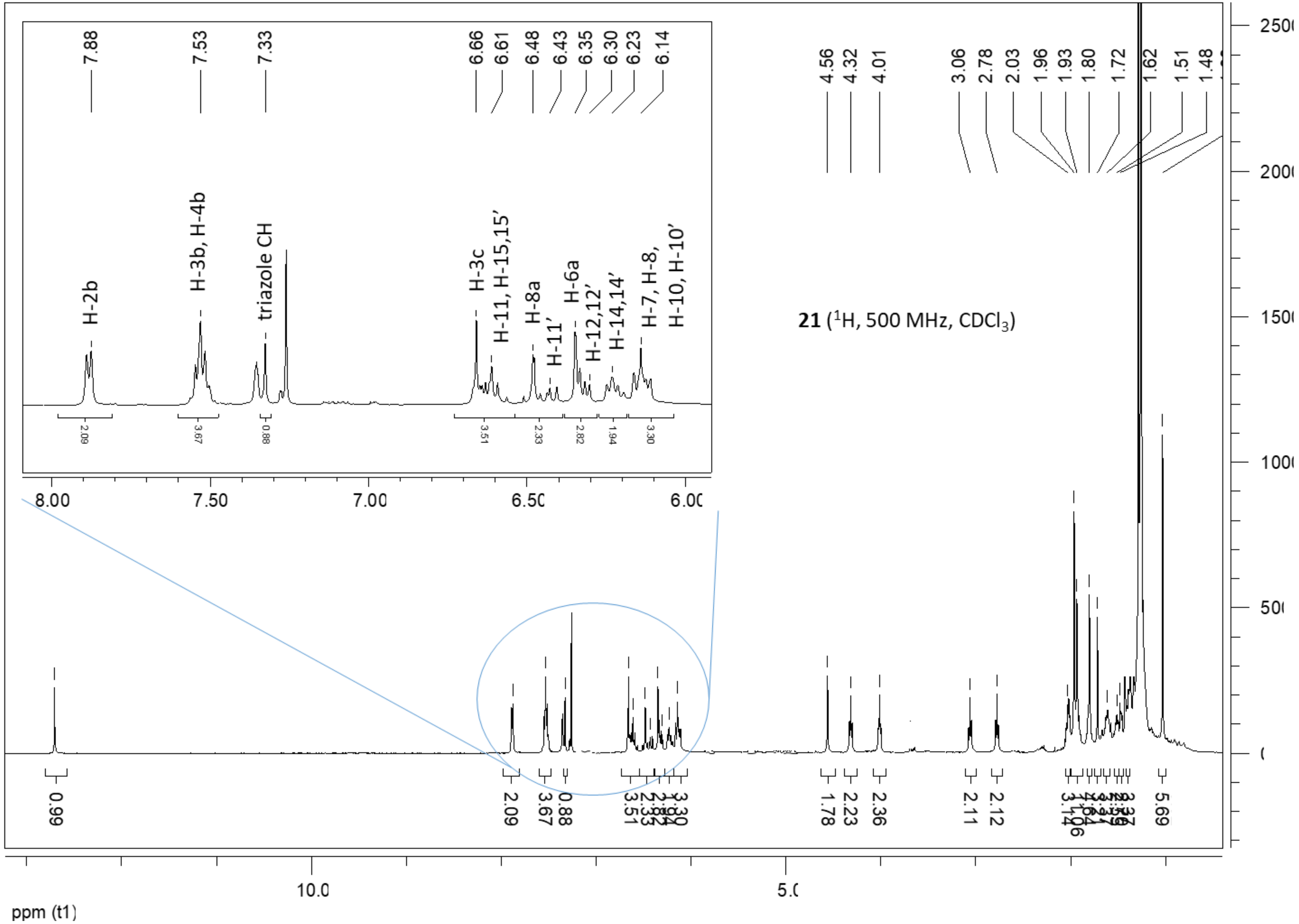

Figure S32.b. ^{1}H NMR spectrum of chrysin-8′-apo-β-carotenol conjugate (**21**)

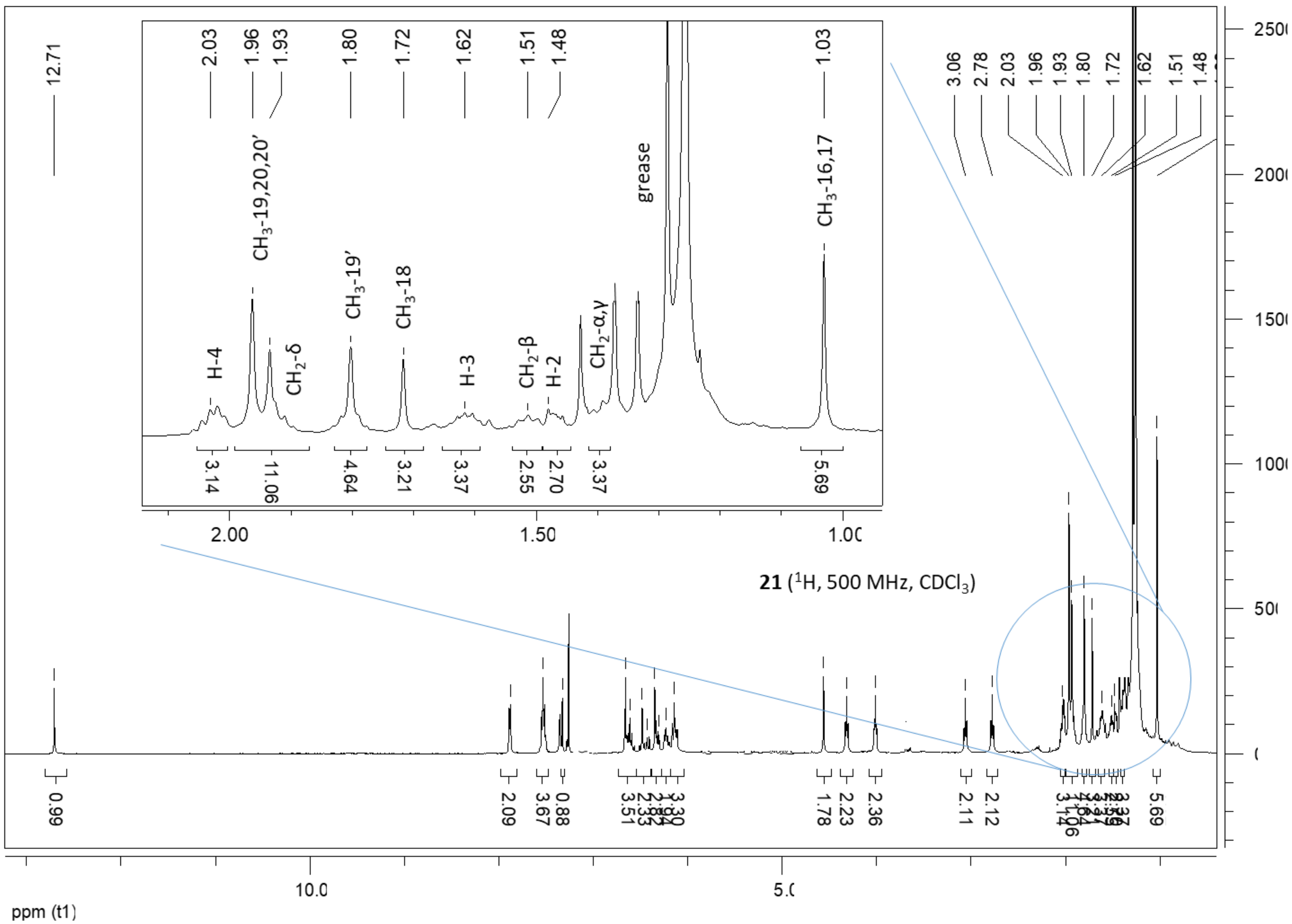

Figure S32.c. ^{1}H NMR spectrum of chrysin-8′-apo-β-carotenol conjugate (**21**)

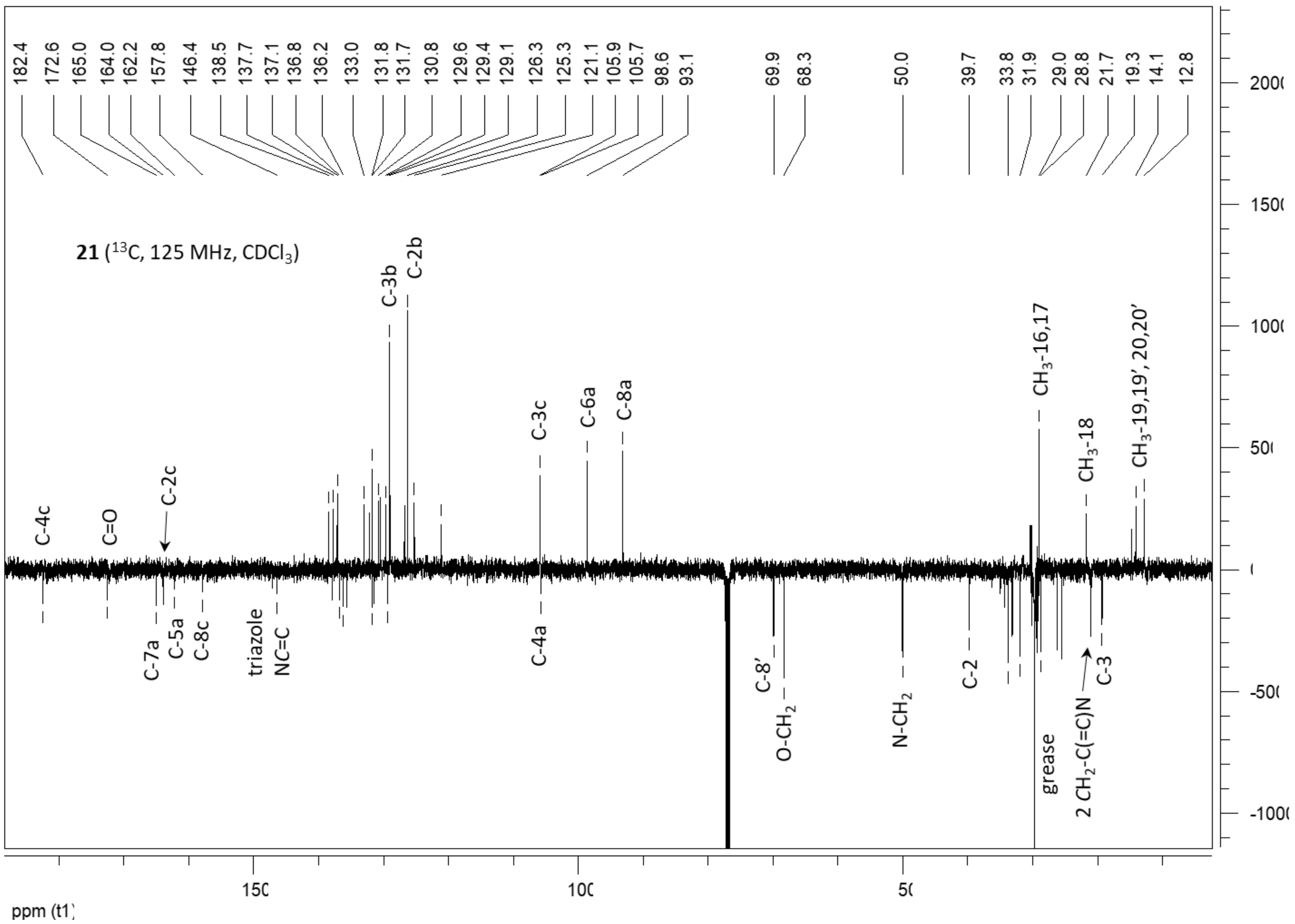

Figure S33.a. ^{13}C-apt NMR spectrum of chrysin-8′-apo-β-carotenol conjugate (**21**)

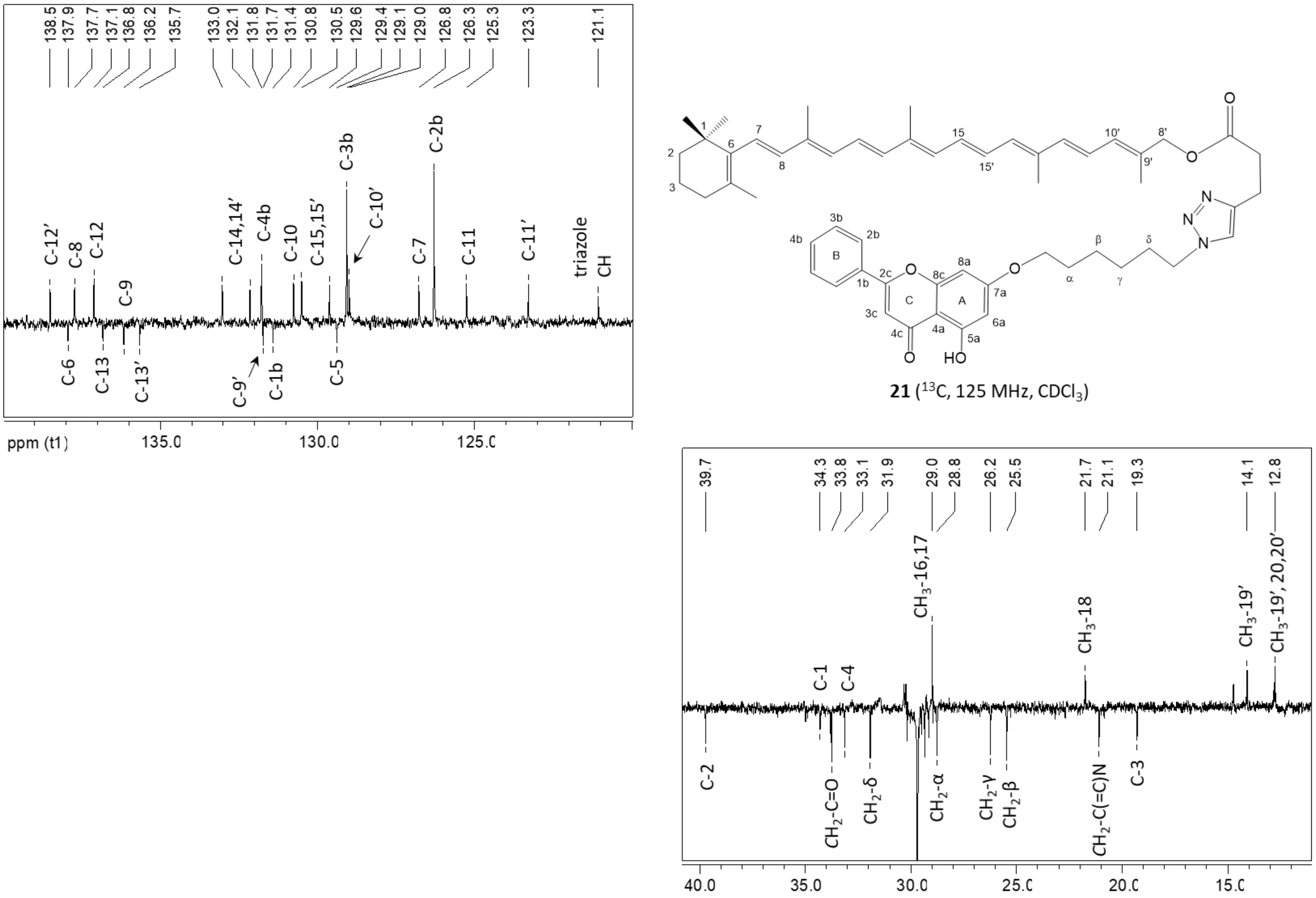

Figure S33.b. ^{13}C-apt NMR spectrum of chrysin-8′-apo-β-carotenol conjugate (**21**)

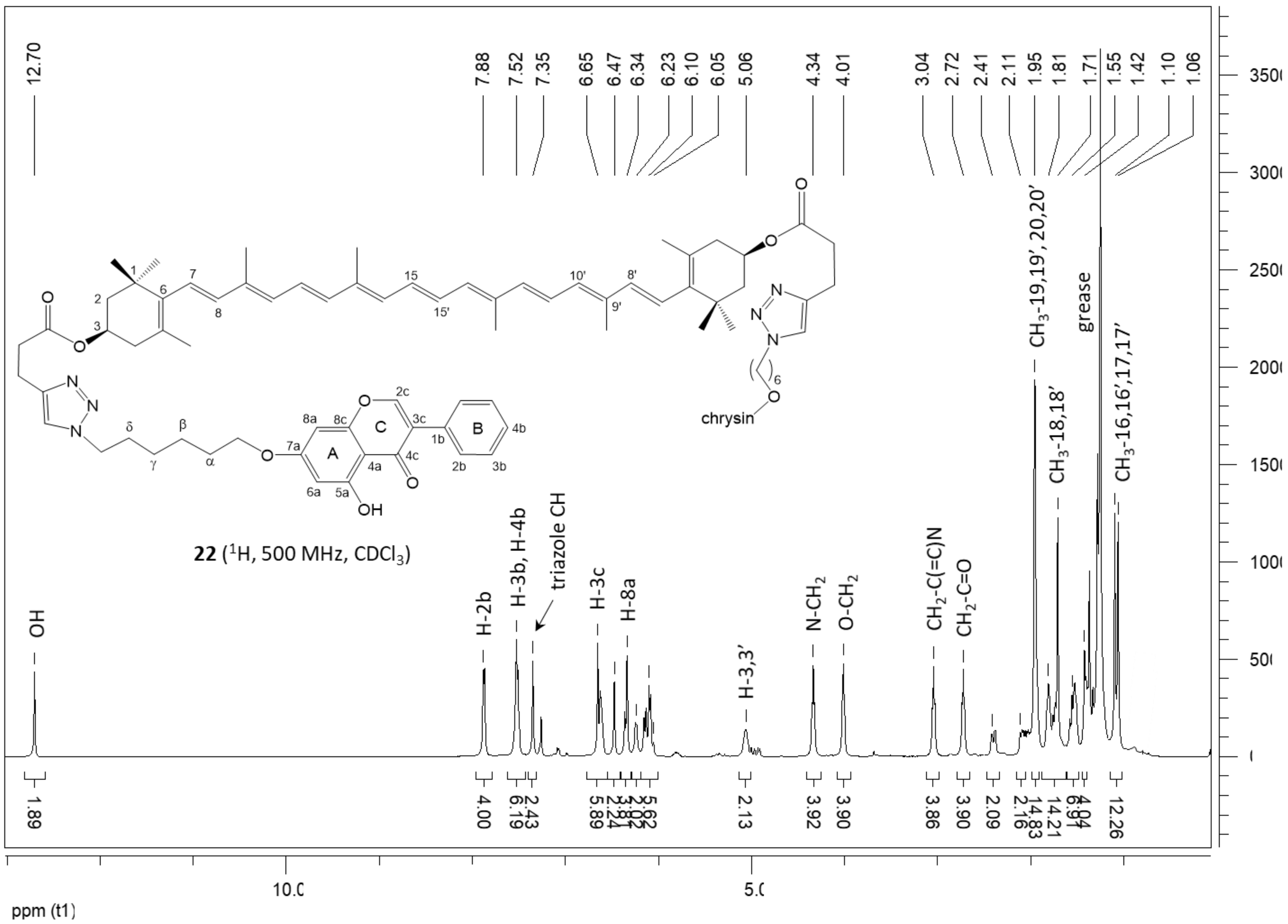

Figure S34.a. ^{1}H NMR spectrum of *bis*-chrysin-zeaxanthin conjugate (**22**)

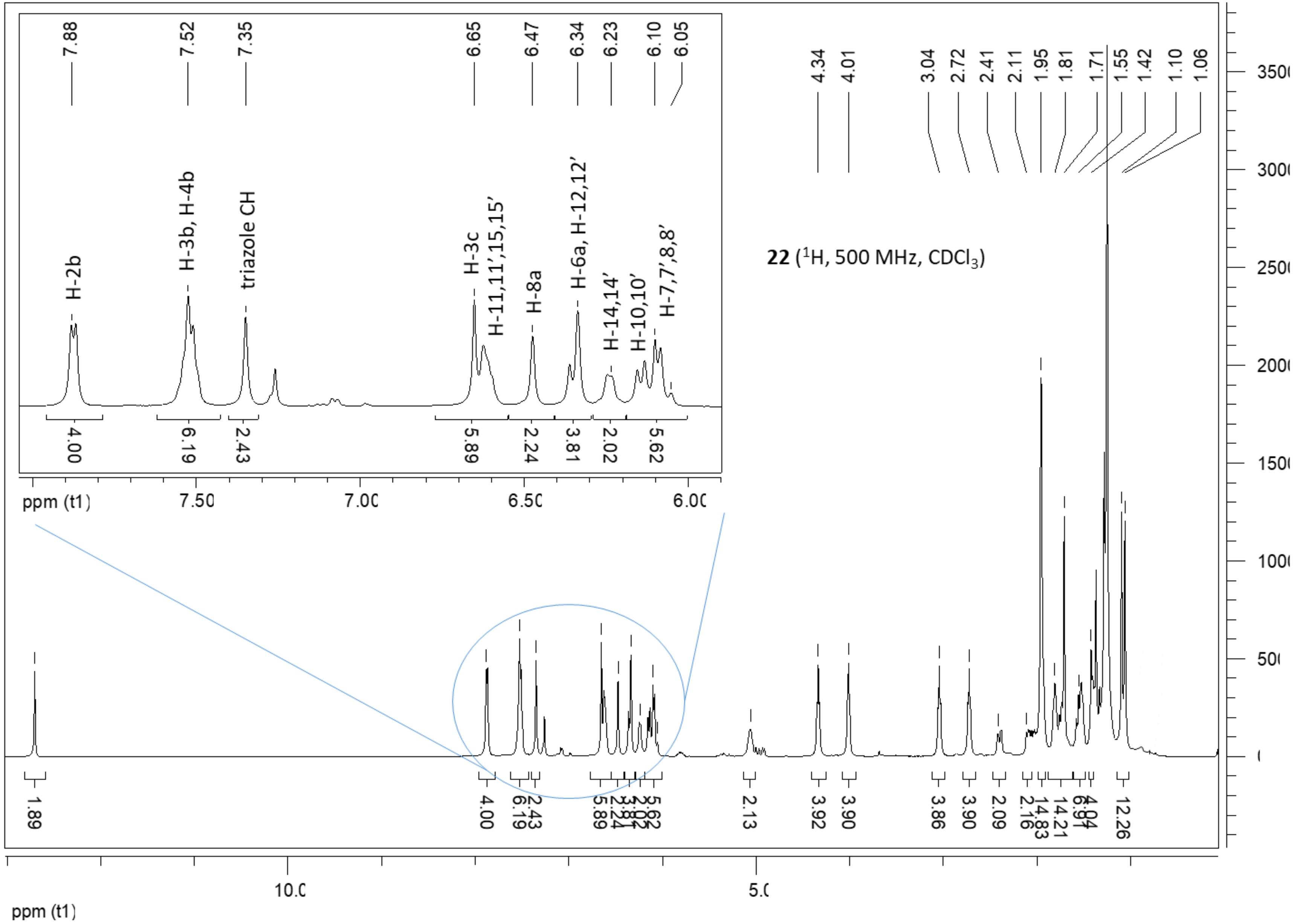

Figure S34.b. ^{1}H NMR spectrum of *bis*-chrysin-zeaxanthin conjugate (**22**)

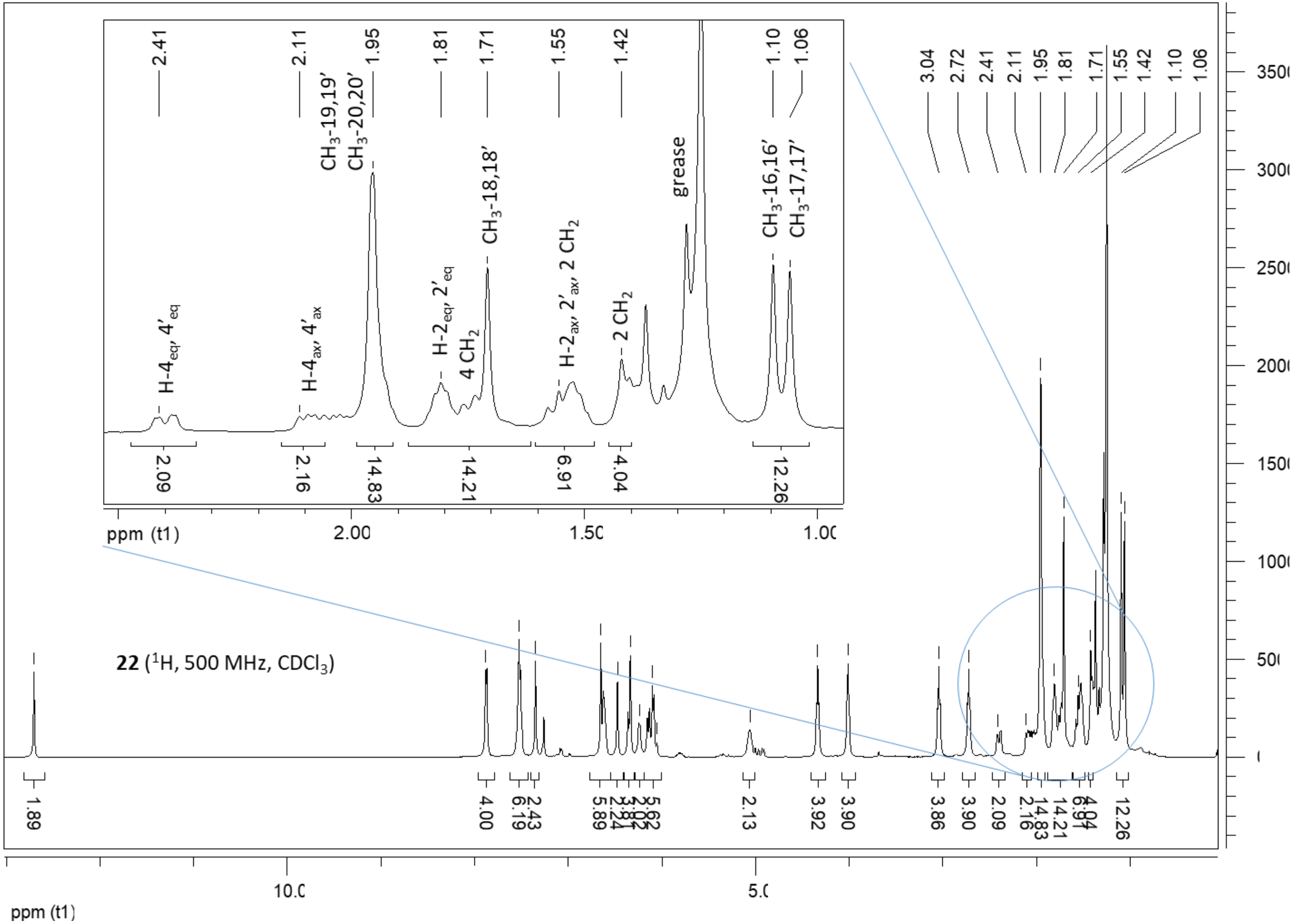

Figure S34.c. ^{1}H NMR spectrum of *bis*-chrysin-zeaxanthin conjugate (**22**)

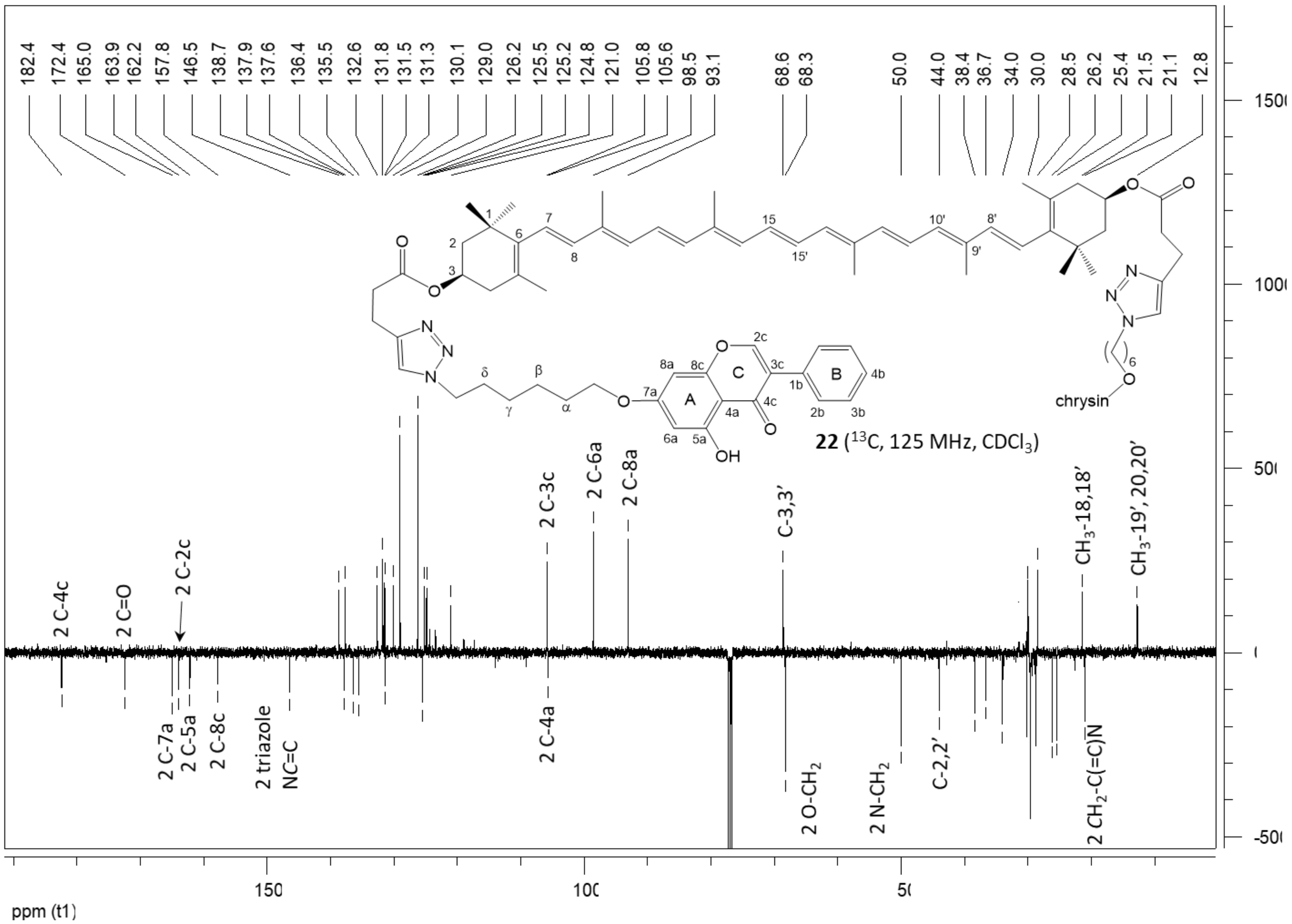

Figure S35.a. ^{13}C-apt NMR spectrum of *bis*-chrysin-zeaxanthin conjugate (**22**)

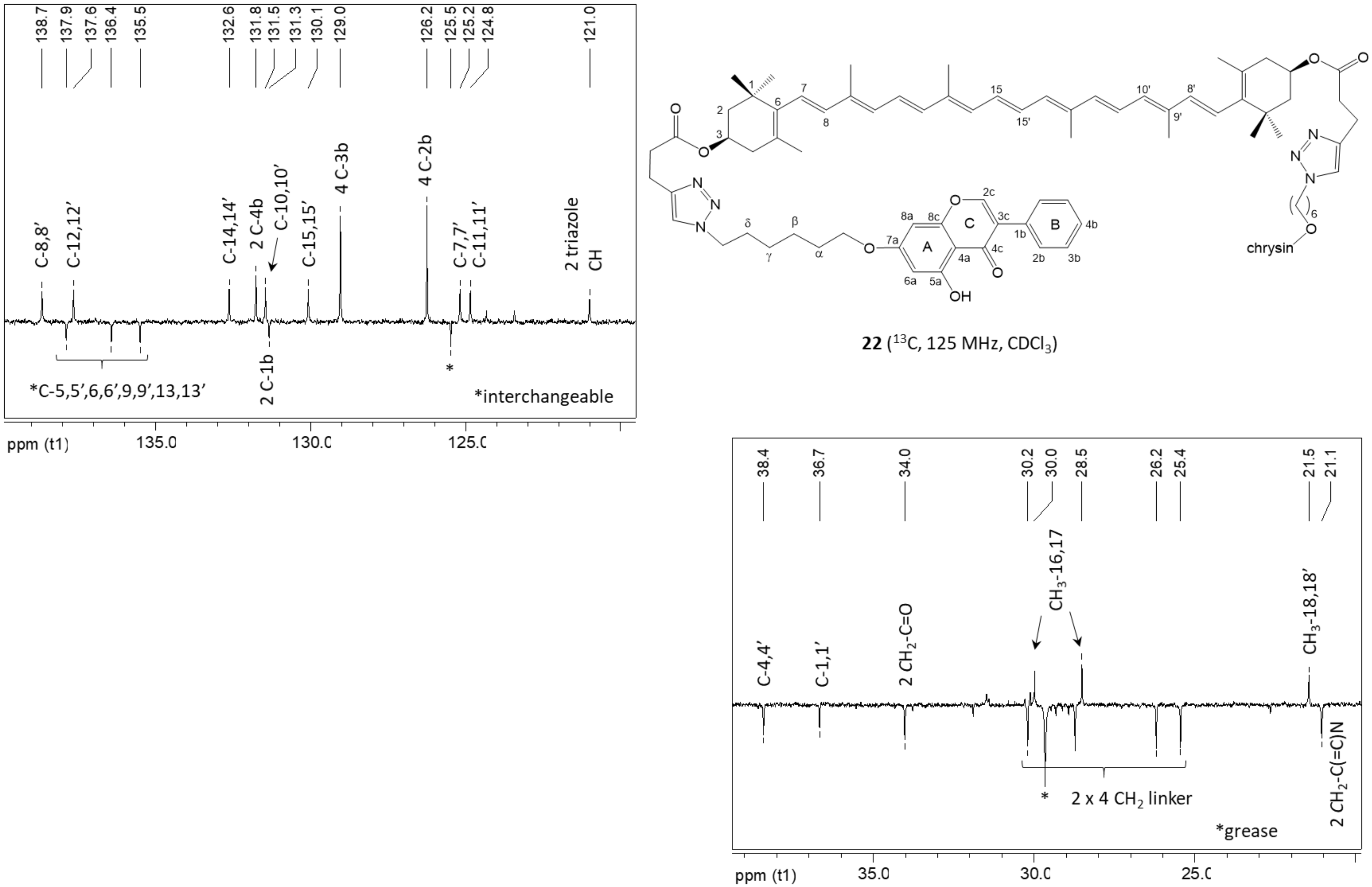

Figure S35.b. ^{13}C-apt NMR spectrum of *bis*-chrysin-zeaxanthin conjugate (**22**)

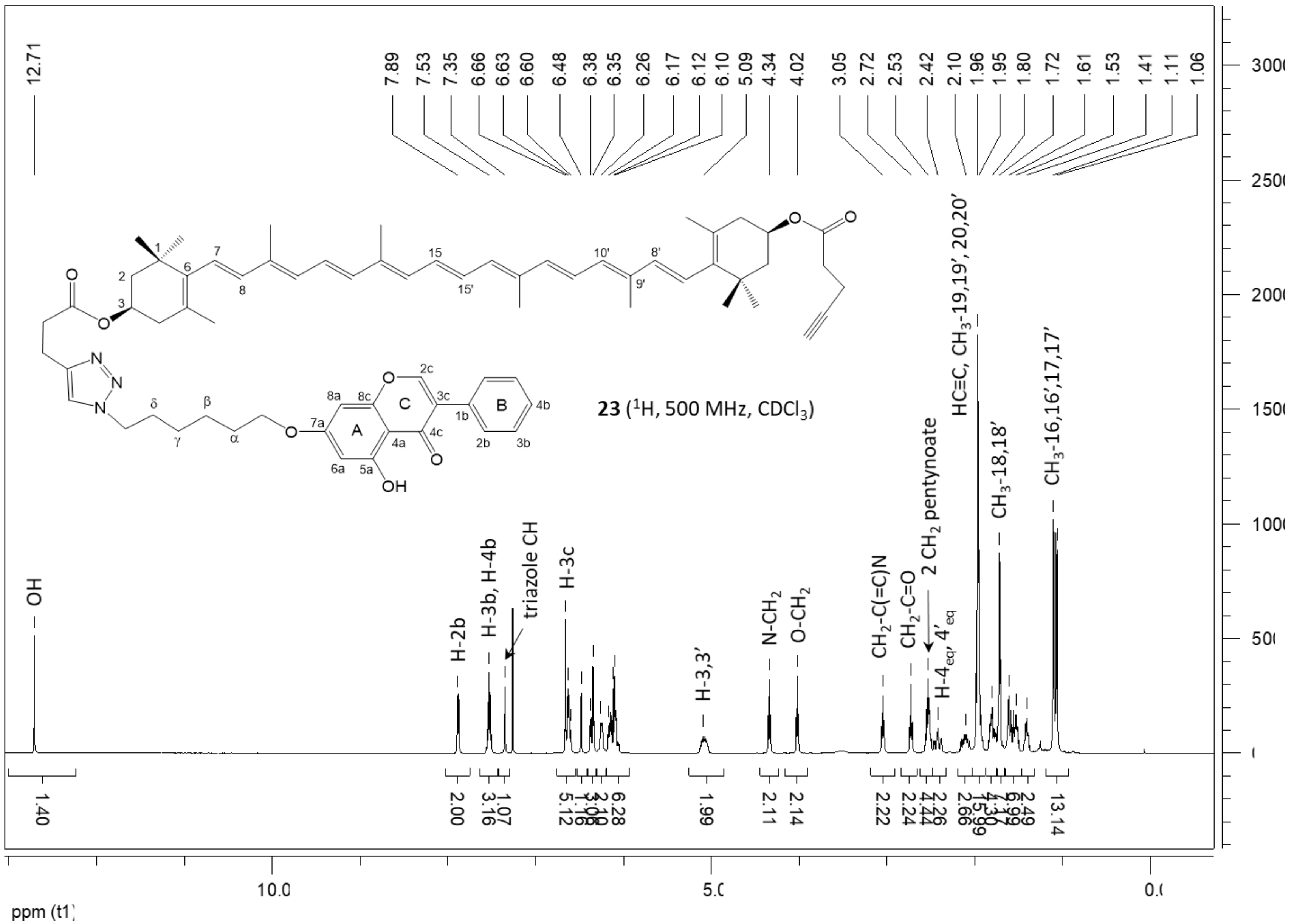

Figure S36.a. ^{1}H NMR spectrum of chrysin-zeaxanthin conjugate (**23**)

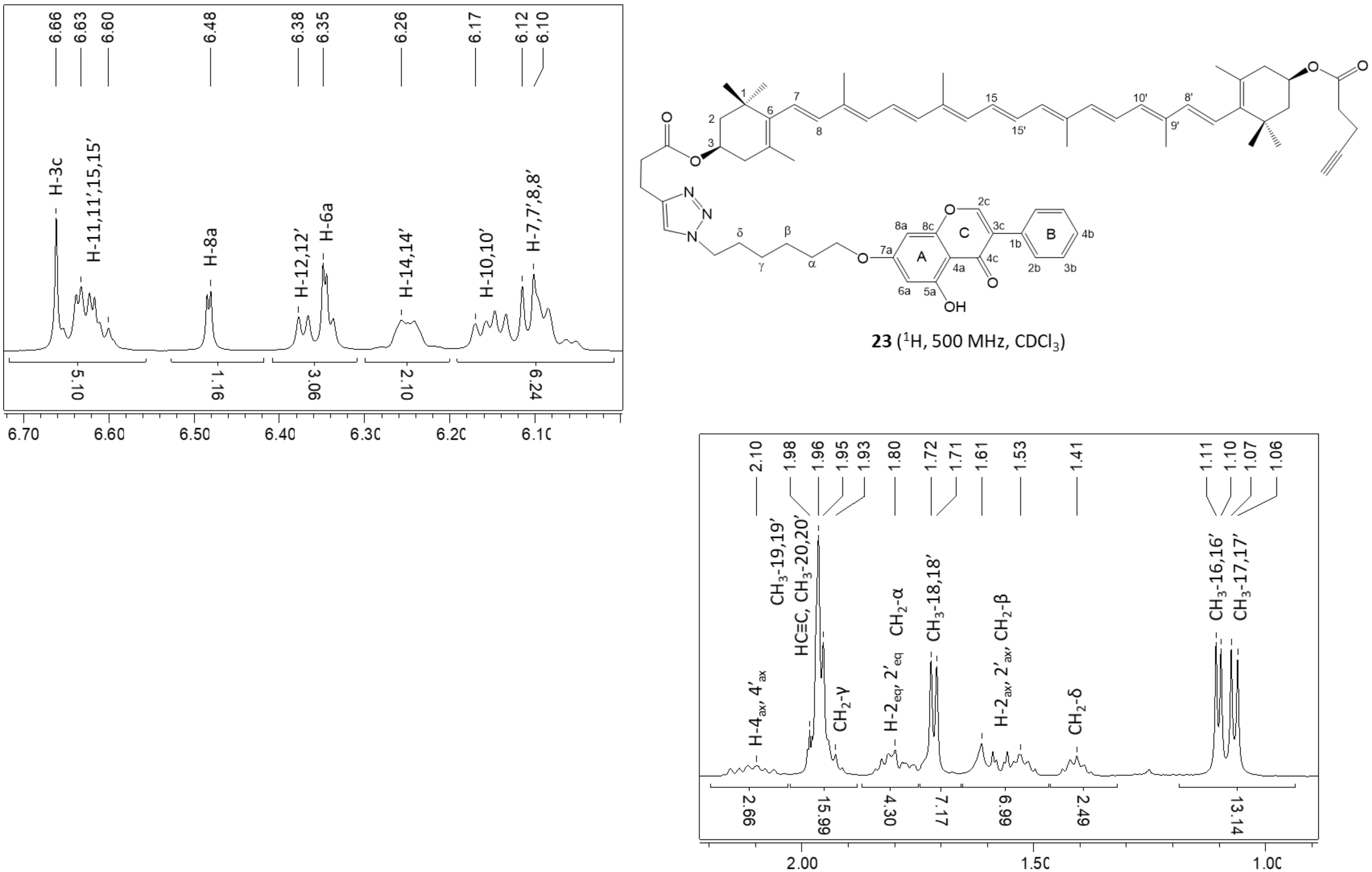

Figure S36.b. ^{1}H NMR spectrum of chrysin-zeaxanthin conjugate (**23**)

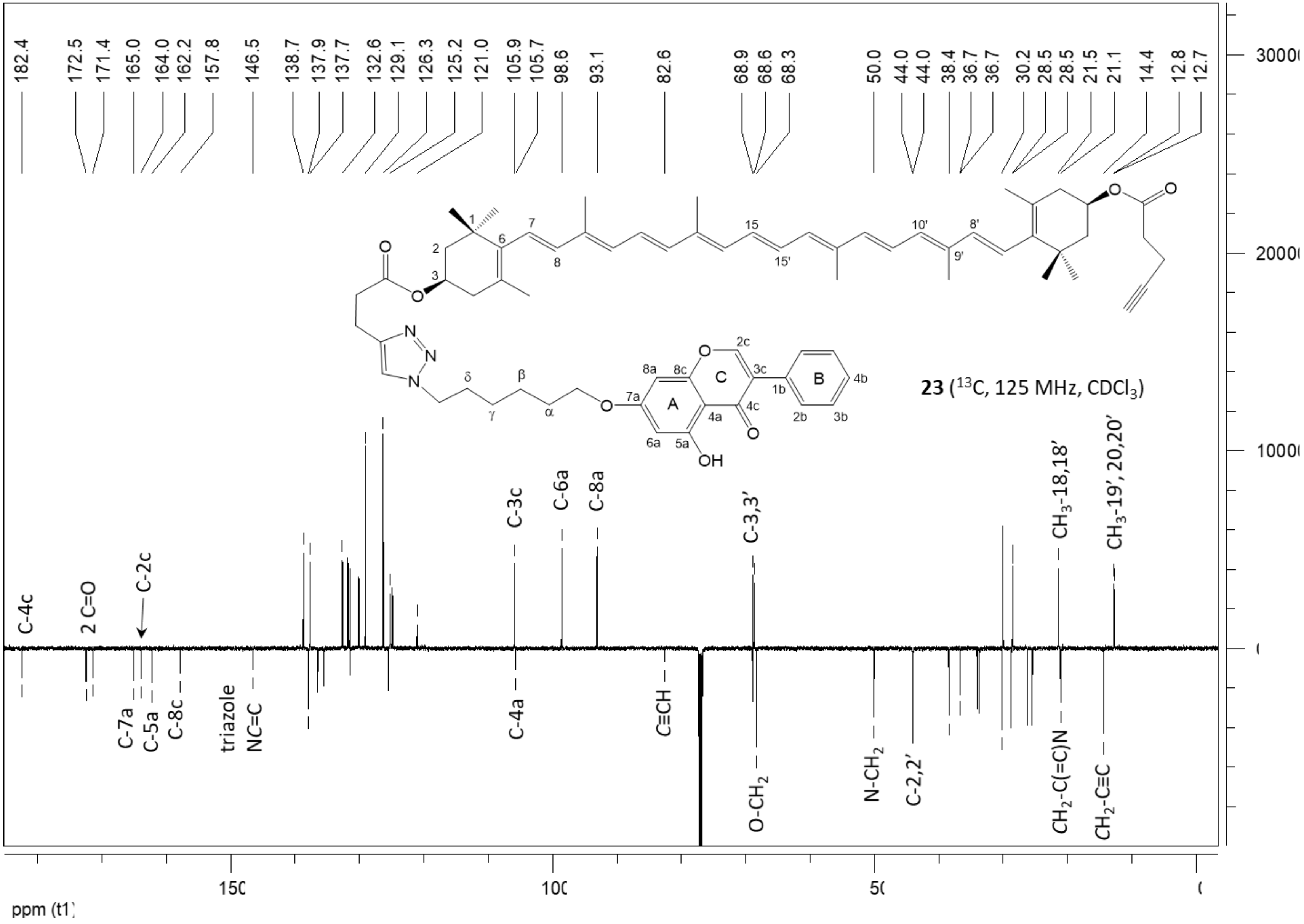

Figure S37.a. ^{13}C-apt NMR spectrum of chrysin-zeaxanthin conjugate (**23**)

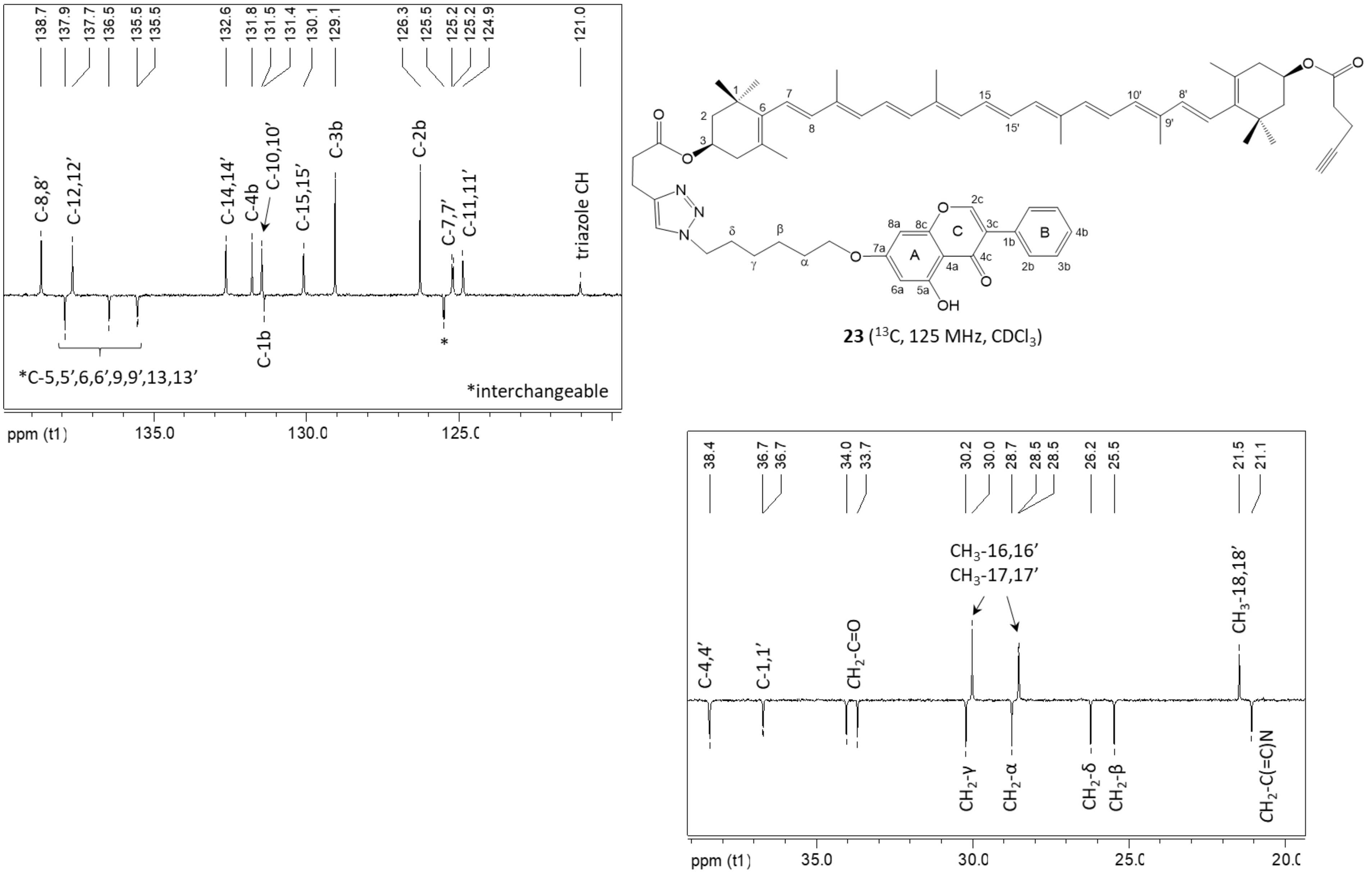

Figure S37.b. ^{13}C-apt NMR spectrum of chrysin-zeaxanthin conjugate (**23**)

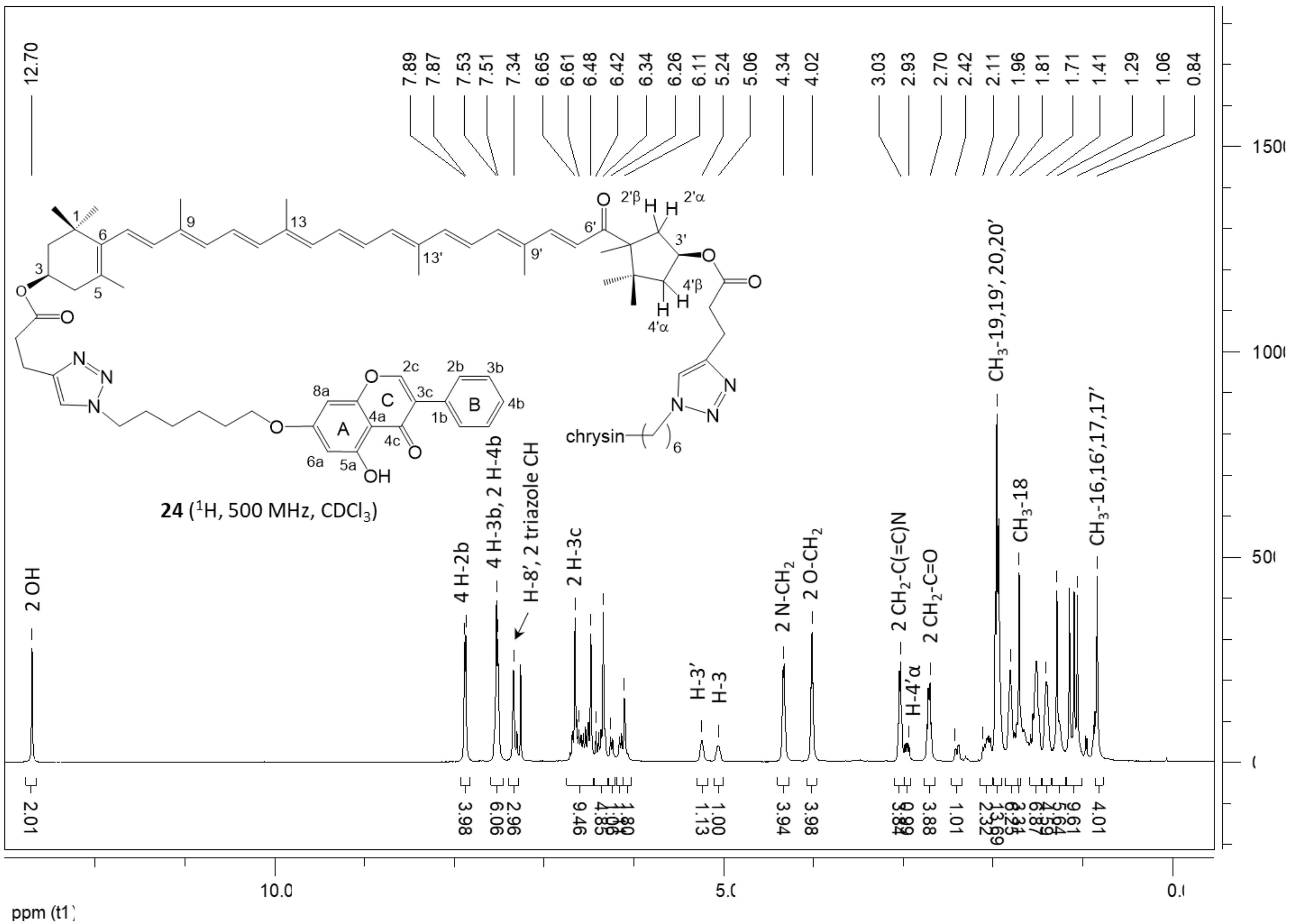

Figure S38.a. ^{1}H NMR spectrum of *bis*-chrysin-capsanthin conjugate (**24**)

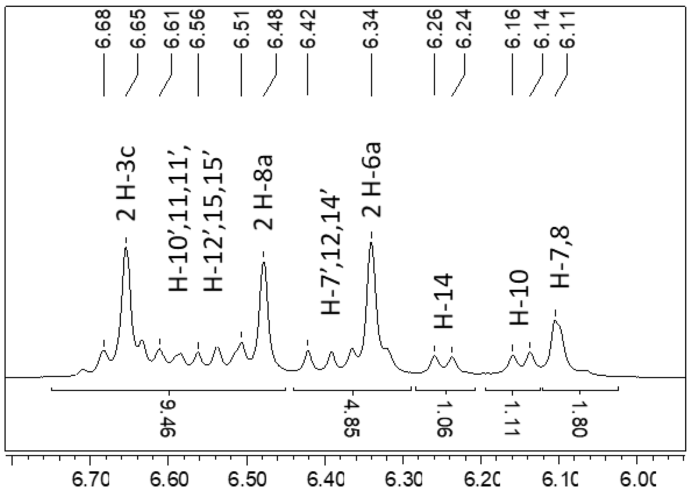

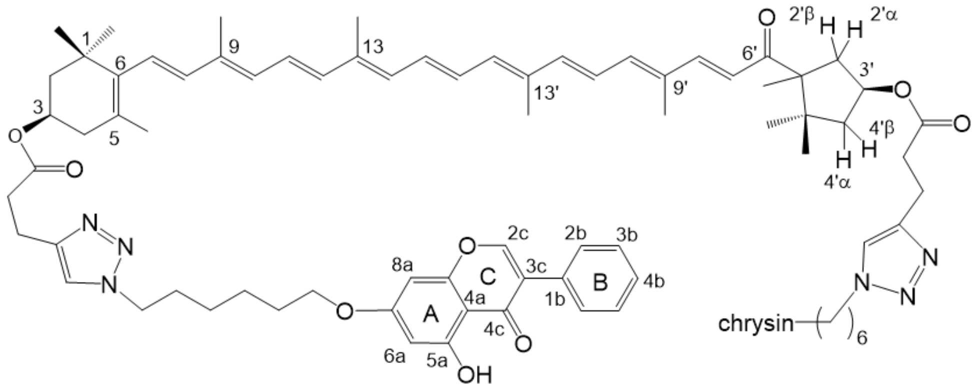

24 (^1H, 500 MHz, $CDCl_3$)

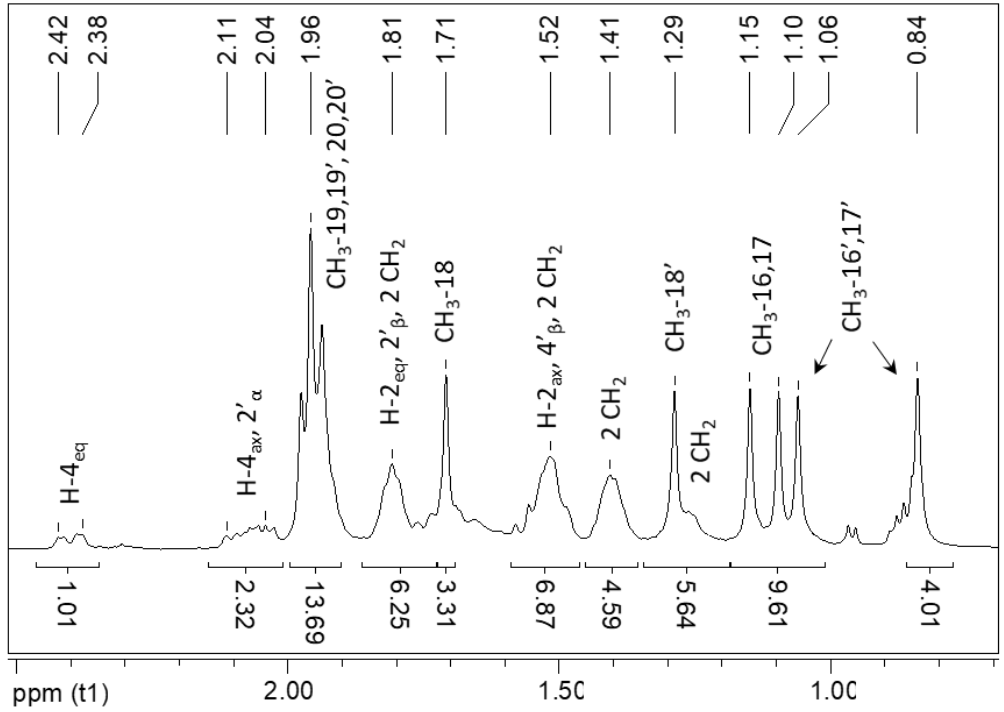

Figure S38.b. ^1H NMR spectrum of *bis*-chrysin-capsanthin conjugate (**24**)

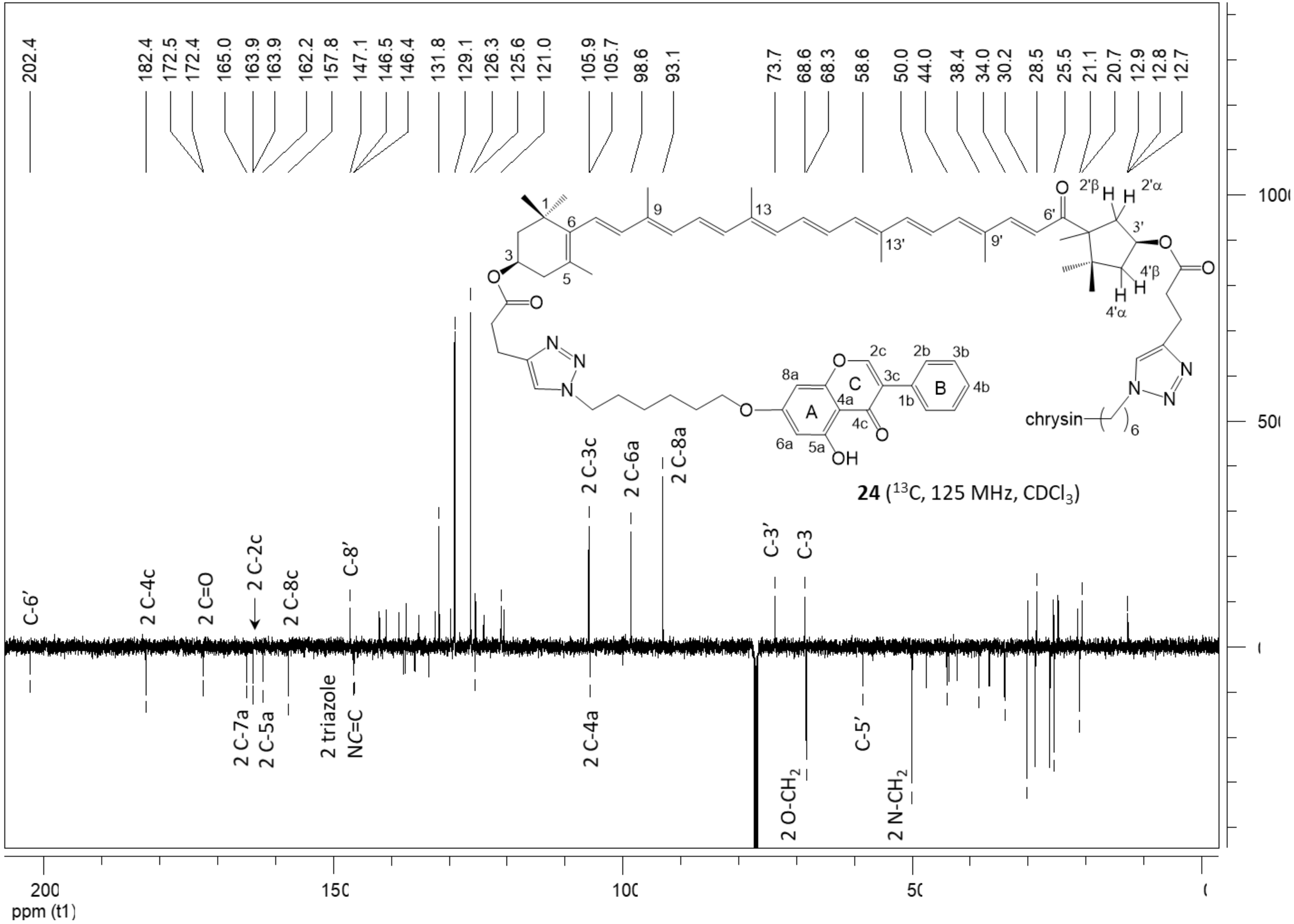

Figure S39.a. ^{13}C-apt NMR spectrum of *bis*-chrysin-capsanthin conjugate (**24**)

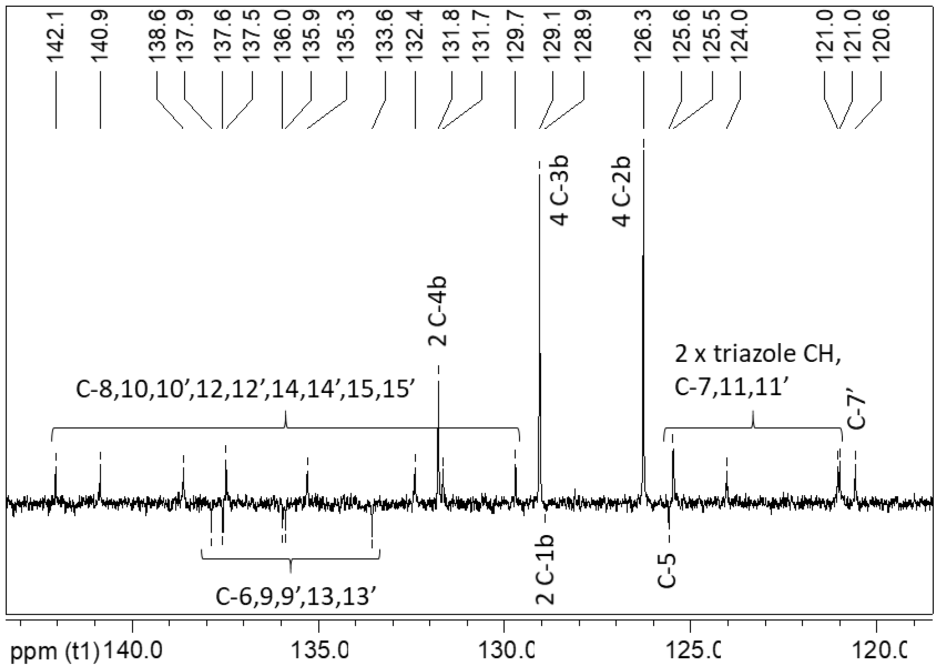

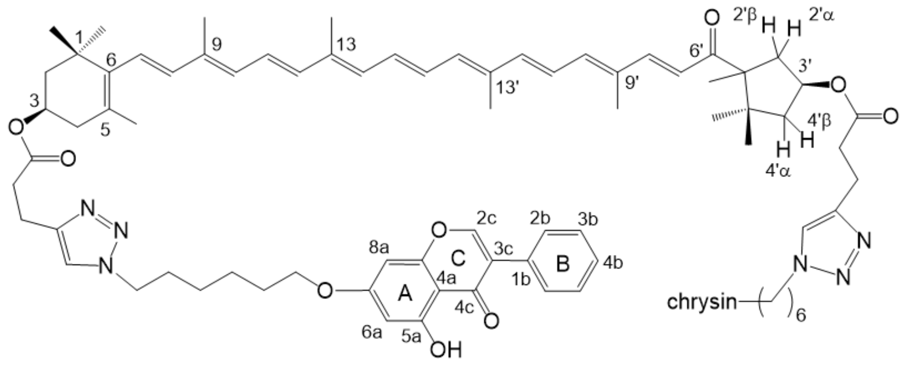

24 (^{13}C, 125 MHz, $CDCl_3$)

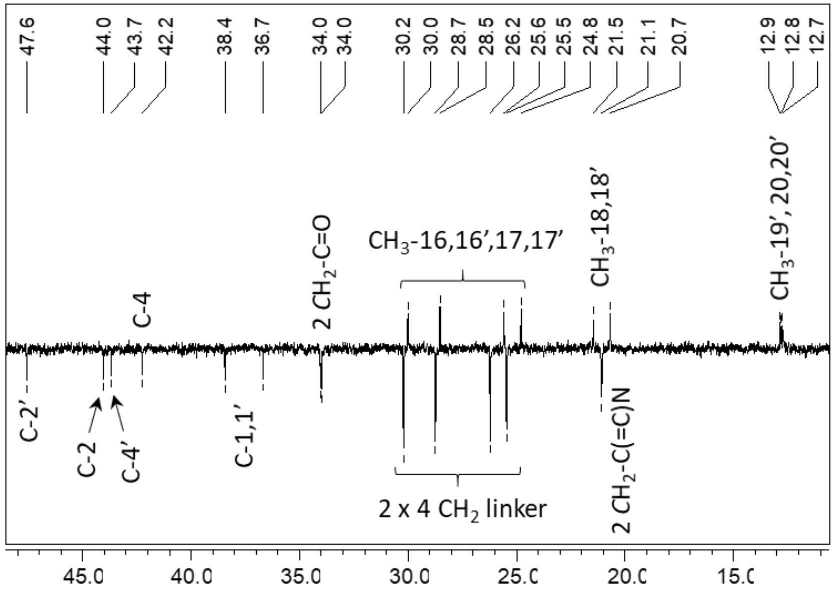

Figure S39.b. ^{13}C-apt NMR spectrum of *bis*-chrysin-capsanthin conjugate (**24**)

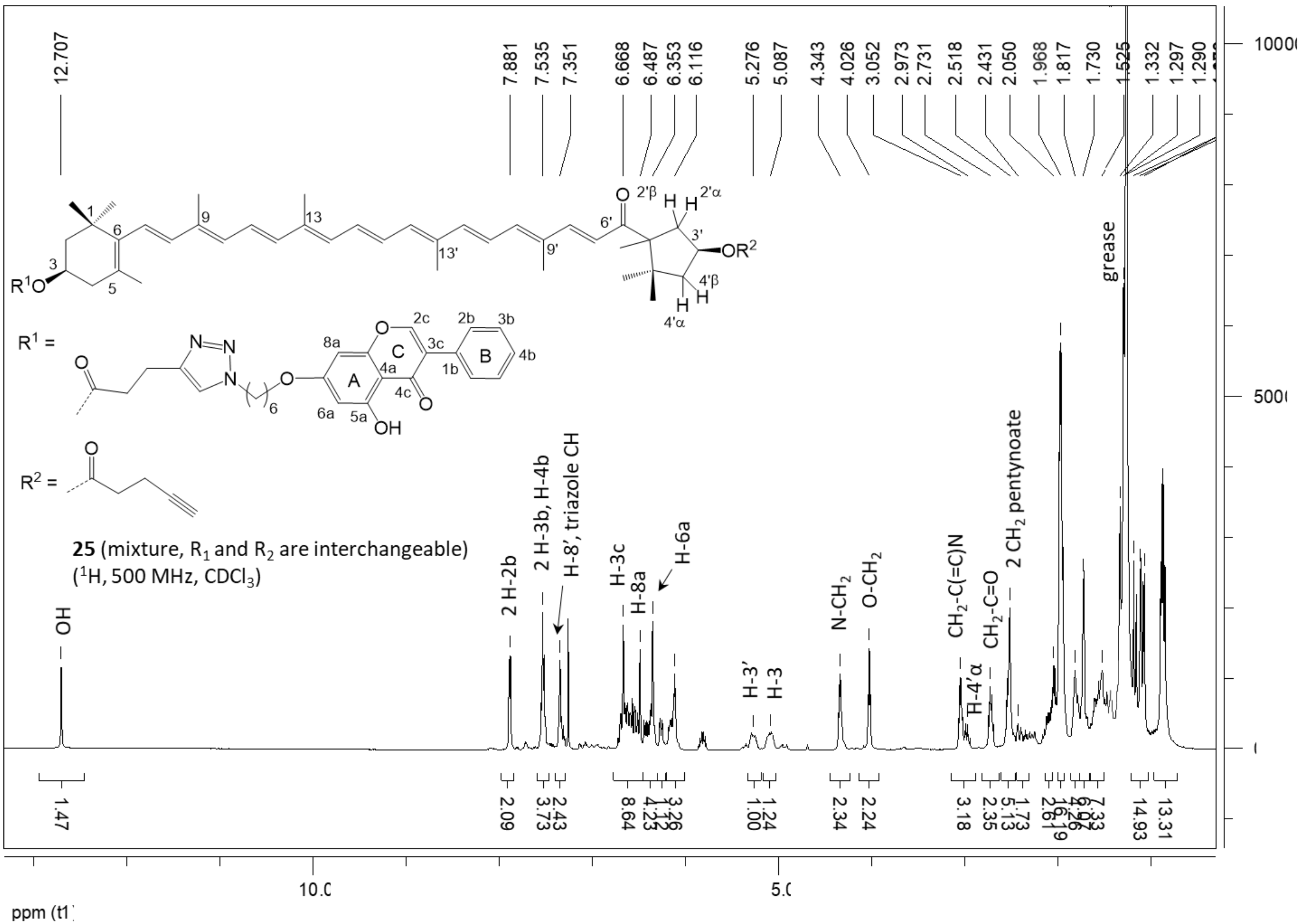

Figure S40.a. ^{1}H NMR spectrum of the mixture of regioisomers of chrysin-capsanthin conjugate (**25**)

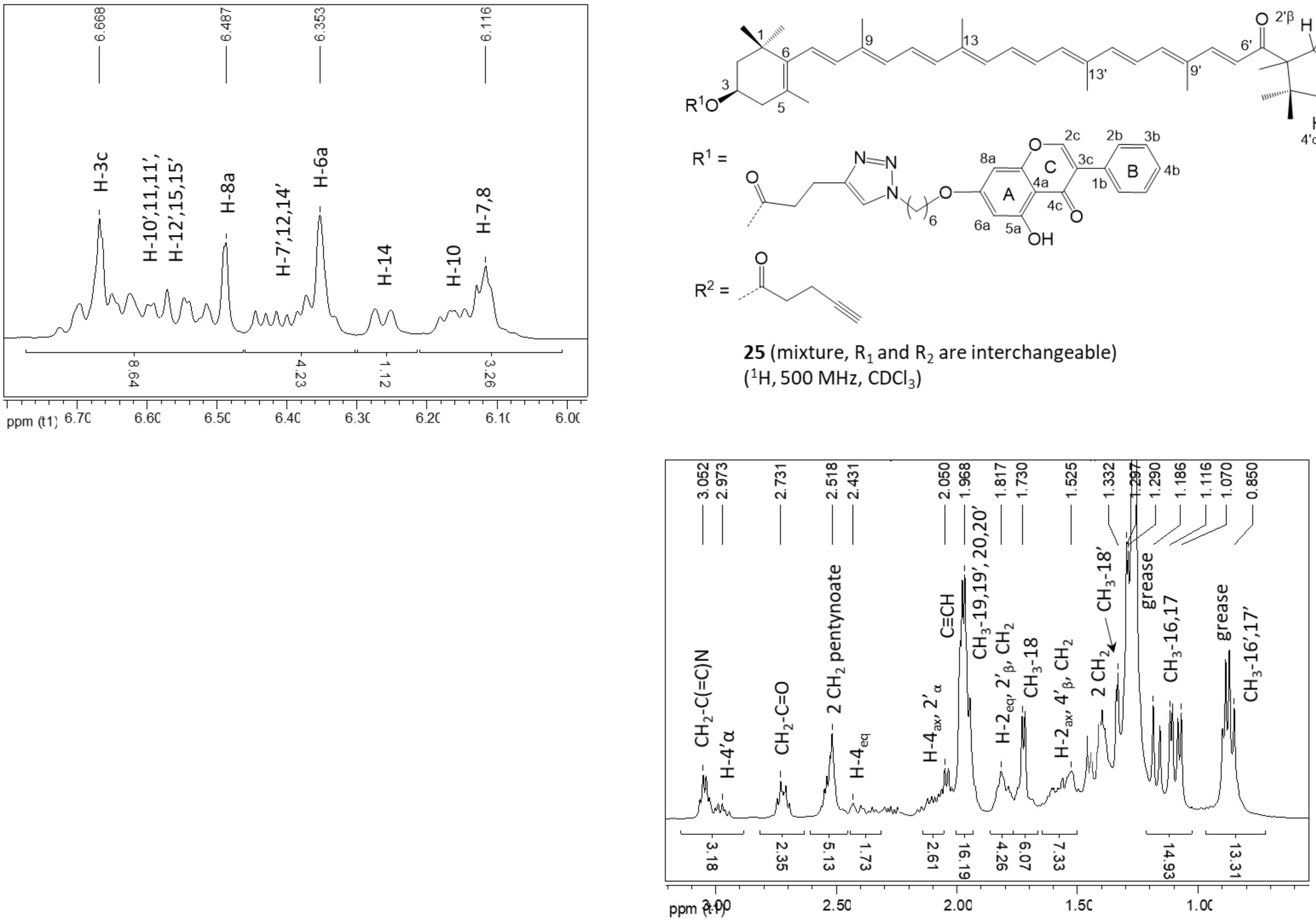

Figure S40.b. ^{1}H NMR spectrum of the mixture of regioisomers of chrysin-capsanthin conjugate (**25**)

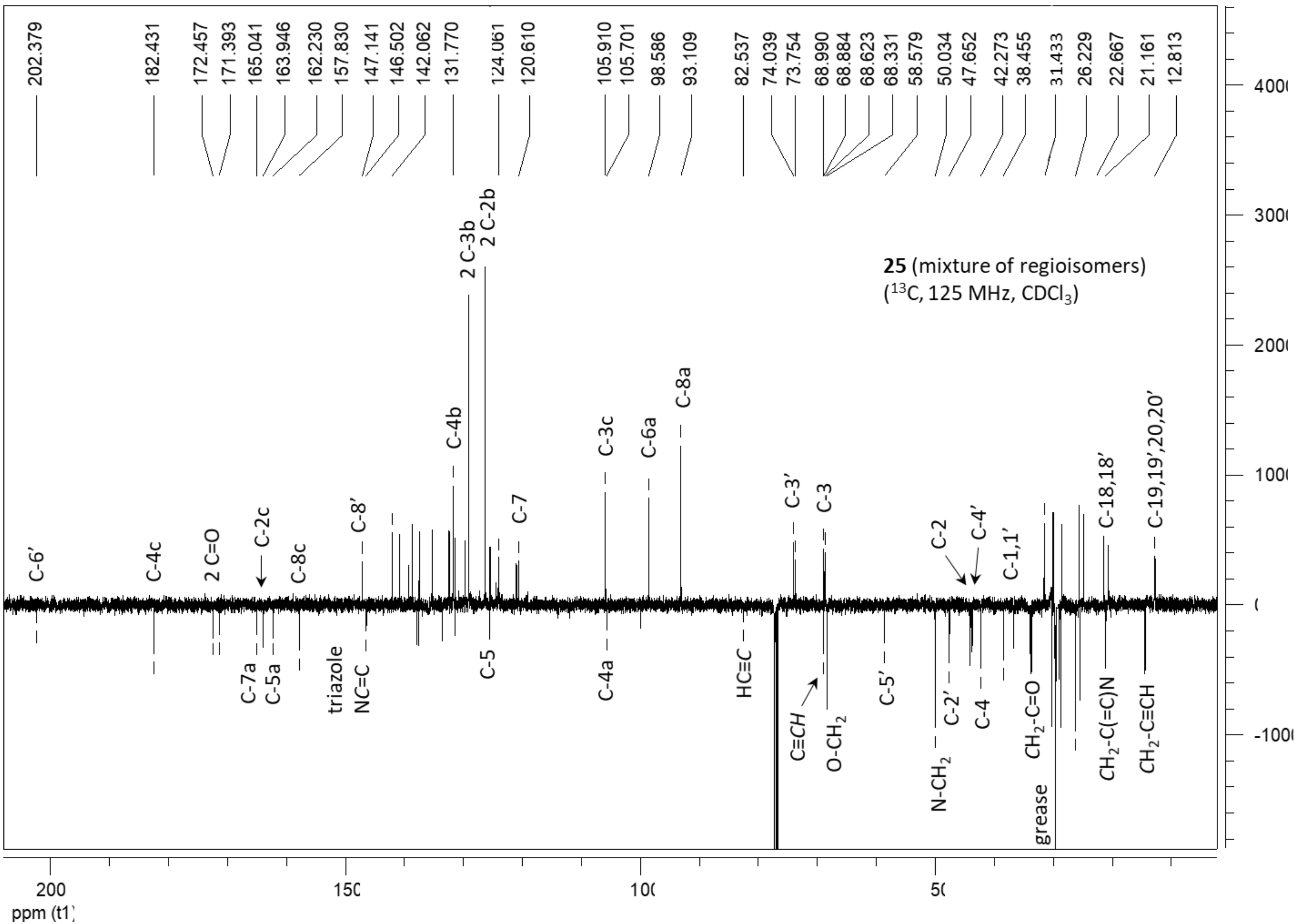

Figure S41. ^{13}C-apt NMR spectrum of the mixture of regioisomers of chrysin-capsanthin conjugate (**25**)
